# Estimated health and economic effects of different salt reduction strategies on cardiovascular disease in Brazil: a microsimulation analysis

**DOI:** 10.1038/s41598-026-49991-1

**Published:** 2026-04-30

**Authors:** Eduardo Augusto Fernandes Nilson, Jonathan Pearson-Stuttard, Brendan Collins, Maria Guzman-Castillo, Simon Capewell, Martin O’Flaherty, Chris Kypridemos

**Affiliations:** 1 Oswaldo Cruz Foundation, Brasilia, Brazil; 2 Universidad Autónoma de Chile, Santiago, Chile; 3 Faculty of Medicine, School of Public Health, Imperial College London, London, UK; 4 Department of Public Health, Policy & Systems, University of Liverpool, Liverpool, UK

**Keywords:** Sodium, Sodium reduction, Sodium targets, Health economics, Cardiovascular disease, Hypertension, Food policy, Public health, Global health, Cardiology, Diseases, Health care, Medical research, Risk factors

## Abstract

Excessive salt consumption is an important risk factor for cardiovascular diseases (CVD), especially through increased blood pressure. In Brazil, the average salt intake is almost twice the international recommendations, and current population-level salt-reduction policies include only voluntary maximum sodium targets for packaged foods and awareness strategies. This study aims to quantify the potential health and economic impacts of additional salt-reduction strategies in Brazil from 2019 to 2038. We used a previously validated microsimulation of a close-to-reality synthetic population (IMPACT_*NCD-BR*_) to estimate CVD events (cases and deaths) prevented or postponed and the corresponding cost savings from 2019 to 2038, stratified by age and sex. The policy scenarios modelled were 1) maintaining the voluntary limits for maximum sodium content in packaged foods; 2) enforcing mandatory lower limits for maximum sodium content in packaged foods; 3) mandatory front-of-pack labelling warnings for excessive sodium in foods, in addition to scenario 1; and 4) universal use of 10% potassium table salt, in addition to scenario 1. Model inputs were informed by nationally representative health and food consumption surveys and high-quality meta-analyses. The cost analysis incorporated the costs of CVD treatment to the National Health System and the costs of informal care. The study found that from 2019 to 2038, maintaining the current voluntary targets could prevent or postpone some 280,000 CVD case-years (95% UI: 140,000 to 490,000) and 5,700 CVD deaths (95% UI: 2,500 to 9,500). Mandatory limits on the salt content of packaged foods could prevent or postpone 590,000 CVD case-years (95% UI: 260,000 to 1,000,000) and 12,000 CVD deaths (95% UI: 5,900 to 19,000). During the same period, mandatory front-of-pack labelling warnings, in addition to the current voluntary targets, could prevent or postpone approximately 420,000 CVD case-years (95% UI: 190,000 to 740,000) and some 8,400 CVD deaths (3,300 to 14,000). The replacement of regular table salt with 10% potassium salt, in addition to the current voluntary targets, could prevent or postpone approximately 870,000 CVD case-years (400,000 to 1,400,000) and 63,000 CVD deaths (31,000 to 96,000). The estimated cost savings related to CVD treatment from implementing the four scenarios were approximately Int$ 0.8 billion (Int$ 0.4b to 1.4b), Int$ 1.7b (Int$ 0.7b to 3.0b), Int$ 1.2b (Int$ 0.5b to 2.2b), and Int$ 2.2b (Int$ 1.1b to 3.9b), respectively. The current Brazilian voluntary sodium targets for packaged foods may yield modest health and economic impacts compared with other strategies. Our analysis suggests that stricter regulatory salt targets for the food industry, mandatory front-of-pack warning labels for excessive sodium in foods, and universal use of low-sodium table salt could substantially improve the health and economic benefits of salt reduction for the Brazilian people and help achieve the World Health Organization goals for salt intake more quickly and more effectively.

**Supplementary Information:**

The online version contains supplementary material available at 10.1038/s41598-026-49991-1.

## Introduction

Unhealthy diets are a leading driver of non-communicable diseases (NCDs). Among dietary risk factors, excessive salt consumption is associated with high blood pressure, cardiovascular diseases (CVDs), gastric cancer and chronic kidney disease^1^. In Brazil, CVDs are the most frequent cause of death among NCDs^2^. The average per capita daily salt consumption of Brazilian adults is 9.3 g, which is almost double the World Health Organization (WHO)^3^ recommended maximum limit of 5 g^4^. The health and economic burdens of excessive salt intake are significant in Brazil, as yearly, approximately 47 thousand deaths from CVDs and over US$ 944 million in direct and indirect costs of CVDs are attributable to excessive salt intake in the country^5^.

Dietary patterns have significantly changed in the country over the last few decades. The traditional diet, based on fresh and minimally processed foods and meals prepared from scratch, has gradually been replaced by processed and ultra-processed foods. As a result, the participation of table salt in the population’s salt consumption has reduced, while the participation of processed and ultra-processed foods has increased over time. Currently, almost 40% of Brazilians’ dietary salt comes from industrialised (processed and ultra-processed) foods, while over 55% comes from table salt.^6^

Hence, the salt reduction policies in Brazil have been based on health education and communication activities aimed at reducing the discretionary use of table salt^7^ and food reformulation strategies that have reduced the salt content of industrialised foods through voluntary upper limits for salt content in priority food categories since 2011^8^.

From 2011 to 2017, food reformulation driven by the national voluntary sodium targets led to a reduction of 8% to 34% in the average sodium content of prioritised packaged foods in Brazil^9^, resulting in an overall 0.25 g/day reduction in population salt intake^10^. However, these voluntary targets haven’t been updated since 2018. We previously estimated that this salt reduction through voluntary sodium reduction targets could prevent or postpone approximately 110,000 CVD case-years and 14,000 total deaths, as well as generate cost savings of US$ 110 million to the National Health System from 2013 to 2032^11^.

The WHO recommended salt reduction through food reformulation as a “best buy” intervention because of its cost-effectiveness and feasibility^12^. Additionally, comprehensive multicomponent strategies involving mandatory food reformulation, food labelling and media campaigns are likely to achieve larger reductions in population-wide salt consumption than individual strategies^13^.

The aim of this study was to estimate the potential health and economic impacts of the regulatory front-of-pack labelling warnings for excessive salt in foods, the universal use of 10% potassium table salt, and regulatory targets for maximum salt content in packaged foods in Brazil from 2019 to 2038.

## Methods

We used a previously validated IMPACT _NCD-BR_ microsimulation for Brazil, based on available local data, to assess the potential health and economic effects of different salt-reduction policy scenarios over 20 years (2019 to 2038).

### Scenarios

In Brazil, from 2019 to 2038 of 1) maintaining the voluntary limits (targets) for maximum sodium content in packaged foods; 2) enforcing mandatory lower limits for maximum sodium content in packaged foods; 3) implementation of regulatory front-of-pack labelling warnings (FOPL) for excessive salt in industrialised (processed and ultra-processed) foods in addition to the existing voluntary limits for maximum sodium content in packaged foods; and 4) universal use of 10% potassium table salt, in addition to the existing voluntary limits for maximum sodium content in packaged foods, compared to a “no intervention” baseline scenario. The second scenario assumed the regulatory implementation of the lowest international targets for maximum sodium content in 2019^14–18^. We further assumed that, given the extent of the reformulation, all individuals were exposed to the policy proportionally to their salt intake from industrialised foods. The impact of FOPL warnings was estimated considering the estimated changes in consumption from the hypothetical implementation of black octagons with the message “high in sodium”^19^ in all foods with over 1 mg of sodium per calorie, as recommended by the Pan American Health Organization (PAHO) Nutrient Profile based on the results of an online survey^20^. The potassium-enriched salt scenario assumed that all table salt consumed in the country contained 10% of potassium chloride. The rationale and more detailed results of the modelled scenarios were included in the Supplementary Materials.

### The IMPACT _NCD-BR_ model

IMPACT_NCD-BR_ is a discrete-time stochastic, dynamic, microsimulation model based on the simulated adult life course of a close-to-reality open cohort of synthetic individuals under different policy scenarios, considering the population heterogeneity and the lag times between exposures and outcomes. The data sources for the model and the model inputs, structure, and key assumptions are detailed in Supplementary Materials Appendix (see S1 Appendix, Tables S1 and S2).

The model first runs the “no intervention” scenario, simulating the life courses of the simulated individuals and recording salt consumption, systolic blood pressure, first cardiovascular episode, and death (from CVD or any other cause). Afterwards, it simulates the life courses of the same population under different intervention scenarios and records their outcomes.

The close-to-reality population used in the model mirrors the Brazilian national population structure by age and sex^21^, including data from the National Health Survey (*Pesquisa Nacional de Saúde—*PNS 2013)^22^ and the National Household Budget Survey (*Pesquisa de Orçamentos Familiares—*POF 2008–2009 and 2017–2018)^23–26^ regarding salt and systolic blood pressure (SBP) exposure.

### Microsimulation model structure

**Fig. 1:**
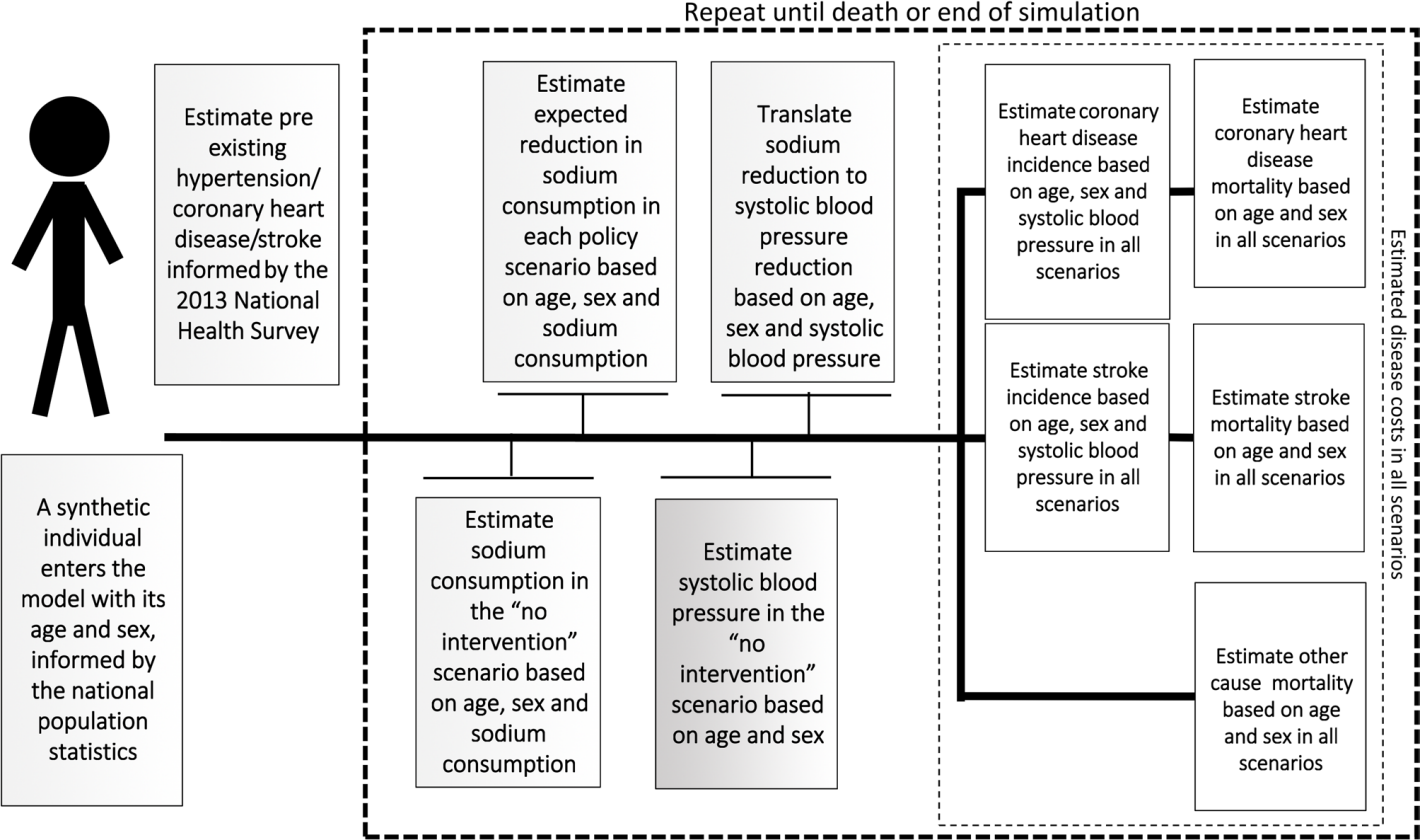
Simplified Structure of the Brazil IMPACT sodium policy model.

The model tracks individual‐level salt consumption, accounting for dietary salt sources, their impact on SBP, and the consequent risk of CHD, stroke, and finally, death from any cause. IMPACT_NCD-BR_ is calibrated to forecasts of CHD, stroke, and any other-cause mortality for the Brazilian population from 2019 to 2038. The results are presented for adults aged 30 to 79 years over a 20-year simulation horizon (Fig. 1).

The use of a microsimulation model such as the IMPACT_NCD_ microsimulation framework allows for greater flexibility and more detailed simulation. Additionally, it may include different lag times between exposures and outcomes as well as gradients in risk factor trends and has been used in different countries, including Brazil.

For this, the model draws the traits of the simulated individuals from conditional distributions, projects the future sodium intake and uses these projections to evolve the traits of the synthetic individuals over time. We used data from the 2013 National Health Survey for the SBP projections (assuming SBP remains constant over time for all age and sex groups) and POF 2008–2009 and 2017–2018 for the salt intake projections^22–24^.

### Summary of evidence regarding the risks of excess sodium consumption

Excess dietary sodium consumption has been linked to an increased risk of CVD^27^. For CVD, the excess risk appears to be mainly mediated through the deleterious effect of excess sodium consumption on blood pressure (BP)^28^. Our methods for evaluating the causality of sodium reduction on BP and BP reduction on CVD have been previously described^28^.

### Policy effects

The sodium target scenarios for processed and ultra-processed foods included maintaining the current voluntary sodium targets (scenario 1) and implementing mandatory targets based on the lowest targets globally implemented as 2019 (scenario 2), using data from official national food labelling surveys in 2017 and 2019 to estimate changes in food composition. For the voluntary target scenario, we have considered changes in food composition from the most recent documented official monitoring^29^, assuming that sodium content was reduced for the targeted food categories (Supplementary Table S3) only by industries that have voluntarily committed to the national sodium targets (which correspond to a 70% market share in the country). For the mandatory sodium target scenario modelling, we selected the lowest targets in force in 2019 for Brazil, England, Canada, the United States, Argentina, and South Africa and the first regional targets set by PAHO^18^ (Supplementary Table S4) and used these values to map the Brazilian food survey data, reestimating the sodium content of all targeted food categories in the market. We assumed that reformulating food products would adjust sodium content to targets in 2019 and that this would lead to an immediate change in salt intake among simulated individuals. We also assumed that the reformulated products would maintain a stable sodium content afterwards.

The regulatory FOPL warnings for excessive sodium in foods (scenario 3) modelled the impact of adopting octagon warnings with messages of “high sodium” in foods, considering the thresholds recommended by the Pan-American Health Organization^28^, and assuming that part of the population would replace foods with warnings with fresh and minimally processed foods according to Khandpur et al.^15^. We assumed that the voluntary limits modelled in scenario 1 also applied in this scenario.

The salt-substitute scenario assumed that all table salt in Brazil was replaced with 10% potassium table salt, recognizing the cardiovascular benefits of sodium reduction and increased potassium intake. The impact of increasing potassium intake was estimated using a comparative risk assessment approach in parallel to the sodium model, considering the meta-analysis by D’Elia et al.^30^ and similar lag times to sodium for the impact on cases and deaths prevented or postponed. We assumed that the voluntary limits modelled in scenario 1 also applied in this scenario.

These data enabled the model to estimate the potential impact of the modelled policies on each simulated individual based on their age, sex, and sodium consumption in the “no intervention” baseline scenario. The model then used the estimated reduction in sodium consumption of the synthetic individuals to calculate the effect upon their SBP using a published meta-regression equation^28^.

We conservatively assumed a median lag of 5 years from a change in SBP to the risk of CVD cases-years and CVD deaths (assumed to be the sum of CHD and stroke case-years and deaths). Changes in sodium intake influence SBP within weeks, hence we assumed no lag^28,31^. Additionally, we assumed no lag time between policy implementation and changes in sodium intake, and full compliance by the food industry across all policy scenarios..

### Modelling the trends in sodium sources

We assumed that the use of table salt in the population was not affected by any of the modelled scenarios. However, we used the publicly available data from the Household Budget Surveys (POF) of 2002–2003^32^, 2008–2009^23^, and 2017–2018^25^ to estimate the changes in dietary salt (non-industrial and industrial sources) between surveys. We projected the continuation of the replacement of discretionary salt (table salt) by processed and ultra-processed foods (representing the other salt sources), assuming that the replacement would continue at the same rate in the future (see S1 Appendix, Table S5-S8).

### Model outputs

The model generates the total number of relevant events, reported case-years (CHD and stroke), and deaths prevented or postponed (CHD, stroke or other) for each scenario. We present results for Brazilian adults aged 30 to 79 years from 2019 to 2038 (a 20-year simulation horizon), rounded to 2 significant digits. For simplicity, we used the term "cases prevented or postponed" to express case-years prevented or postponed (please see S1 Appendix, Table S9).

### Medical costs analyses

The CHD and stroke hospitalisation costs to the Brazilian National Health System (SUS – *Sistema Único de Saúde)* were obtained from the publicly available tables from the Hospital Information System—SIH-SUS^33^. There were no readily available Brazil-specific data for the primary health and informal care costs; therefore, these costs were estimated indirectly, assuming that the proportion of these costs in relation to the costs of hospitalisations would be similar to that found in estimates from a study on European Union (EU) countries^34^. Costs to the health system and the population were estimated considering the CHD and stroke cases prevented or postponed and the average costs for a person-year living with these diseases. Costs were collected in Brazilian Reals (R$) and subsequently converted to International dollars (Int$), considering the purchasing power parities values in 2019, as reported by the Organisation for Economic Cooperation and Development (OECD)^35^, and all costs were discounted at a 3% annual rate.

### Uncertainty analyses

We performed probabilistic sensitivity analyses using a second-order Monte Carlo approach to estimate the uncertainty of different model parameters and population heterogeneity to propagate to the outputs^36^. Uncertainty was based on sources of the sampling errors of baseline sodium intake, baseline SBP, and the relative risk of CHD and stroke based on SBP, the uncertainties around the lowest sodium and SBP exposures below which no excess risk is observed, around the effect of sodium on SBP, around the lag between SBP changes and CVD risk changes, around the true incidence of CHD and stroke, and the uncertainty of mortality forecasts. The output distributions produced through the Monte Carlo approach were summarised by reporting the medians and 95% uncertainty intervals (UIs) using the 2.5% and 97.5% percentiles of the output distribution.

## Results

### Health-related outcomes

**Fig. 2:**
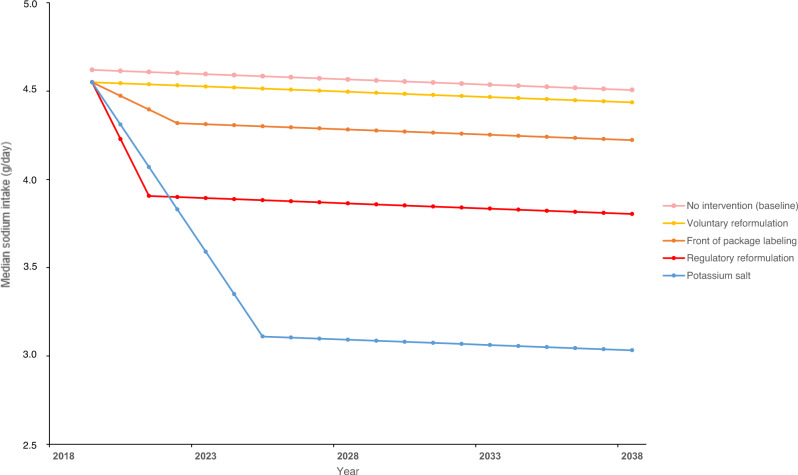
Median daily sodium consumption by year for modelled policy scenarios. Note that the y-axis does not start from 0.

The modelled results suggest that maintaining the voluntary sodium targets would reduce salt consumption by 0.25 g/day, whereas adopting the lowest global sodium reduction targets would lead to a 0.81 g/day reduction. Considering other policies, front-of-pack warnings on excessive salt in foods would reduce salt intake by 0.44 g/day for both men and women, and 10% potassium-enriched table salt would result in a 0.71 g/day reduction for men and a 0.51 g/day reduction for women (Fig. 2).

Furthermore (Table 1), the current national voluntary targets could prevent some.

**Table 1:** Health-related model estimates over a 20-year simulation period, from 2019 to 2038, for Brazilian adults aged over 30 years. Brackets contain 95% uncertainty intervals.

Outcome	Current voluntary salt limits	Mandatory lowest salt limits	Front-of-pack warnings + voluntary limits	10% potassium table salt + voluntary limits
CHD cases prevented or postponed	150,000	310,000	210,000	400,000
	(46,000 to 310,000)	(86,000 to 640,000)	(69,000 to 460,000)	(140,000 to 820,000)
Stroke cases prevented or postponed	130,000	260,000	190,000	440,000
	(49,000 to 270,000)	(94,000 to 580,000)	(68,000 to 410,000)	(170,000 to 790,000)
CVD cases prevented or postponed	280,000	590,000	420,000	870,000
	(140,000 to 490,000)	(260,000 to 1,000,000)	(190,000 to 740,000)	(400,000 to 1,400,000)
CHD deaths prevented or postponed	2,200	4,700	3,200	20,000
	(490 to 4,700)	(1,600 to 9,000)	(480 to 7,300)	(6,700 to 33,000)
Stroke deaths prevented or postponed	3,100	6,500	4,700	43,000
	(1,100 to 5,900)	(3,100 to 12,000)	(1,600 to 8,900)	(16,000 to 70,000)
All deaths prevented or postponed	21,000	52,000	33,000	110,000
	(14,000 to 33,000)	(32,000 to 80,000)	(20,000 to 54,000)	(67,000 to 140,000)

280,000 CVD cases (95% UI: 140,000 to 490,000) and some 5,700 CVD deaths (95% UI: 2,500 to 9,500), as well as 15,000 deaths from other hypertension-related causes (95% UI: 8,700 to 26,000), from 2019 to 2038. Mandatory targets based on the lowest global sodium limits (scenario 2) could prevent some 590,000 CVD cases (95% UI: 260,000 to 1,000,000) and approximately 12,000 CVD deaths (95% UI: 5,900 to 19,000), as well as 40,000 deaths from other hypertension-related causes (95% UI: 22,000 to 66,000) during the same period. Scenario 3 could prevent or postpone some 420,000 CVD cases (95% UI: 190,000 to 740,000), 8,400 CVD deaths (95% UI: 3,300 to 14,000), and 25,000 deaths from other hypertension-related causes (95% UI: 14,000 to 41,000). Finally, scenario 4 could prevent or postpone approximately 870,000 CVD cases (400,000 to 1,400,000) and 63,000 CVD deaths (31,000 to 96,000), as well as 41,000 deaths from other hypertension-related causes (95% UI: 23,000 to 69,000), and front-of-pack warnings for excessive sodium in foods from 2019 to 2038.

The net benefits of salt reduction, especially in reducing CHD, were larger in men than women, reflecting their higher salt consumption and higher CHD and stroke burden. For example, during the 20 years from 2019 to 2038, maintaining the voluntary sodium targets could prevent or postpone approximately 160,000 CVD cases (95% UI: 80,000 to 310,000) in men and some 110,000 case-years in women (95% UI: 51,000 to 220,000), and approximately 3,200 fewer CVD deaths in men (95% UI: 1,500 to 6,400) and 2,200 fewer deaths in women (95% UI: 980 to 4,300), while the mandatory implementation of the lowest sodium targets could prevent or postpone approximately 370,000 CVD case-years (95% UI: 160,000 to 610,000) in men and some 230,000 case-years in women (95% UI: 98,000 to 420,000), and approximately 6,800 fewer CVD deaths in men (95% UI: 3,000 to 12,000) and 4,700 fewer deaths in women (95% UI: 2,200 to 8,200) (please see S1 Appendix, Tables S9-S11).

### Costs of CHD and stroke

From the public healthcare perspective maintaining the voluntary sodium targets could lead to savings of approximately Int$ 0.8 billion (95% UI: Int$ 0.3 to 1,4 billion) in total hospitalisation, primary healthcare, outpatient, pharmaceutical, and informal care costs over 20 years. Most savings would stem from CHD (~ 73%) rather than stroke. Around 76% of the savings are due to reduced CHD and stroke treatment costs for the Brazilian National Health System, with the remaining 24% attributed to savings in informal care costs (Table 2).

**Table 2:** Impact inventory and cost analysis of CVD-related model outputs for individuals aged 30 to 79 years, assessed cumulatively over the 20-year simulation period from 2019 to 2038 (Int$ millions). Brackets contain 95% uncertainty intervals.

Outcome	Current voluntary salt limits	Mandatory lowest salt limits	Front-of-pack warnings + voluntary limits	10% potassium table salt + voluntary limits
Total CHD-related costs	580 (180 to 1,200)	1,200 (340 to 2,500)	830 (270 to 1,800)	1,600 (570 to 3,200)
CHD medical costs to SUS	440 (140 to 920)	940 (260 to 1,900)	640 (210 to 1,400)	1,200 (430 to 2,500)
CHD informal care costs	140 (43 to 280)	290 (80 to 600)	200 (64 to 420)	380 (130 to 760)
Total stroke-related costs	190 (73 to 400)	390 (140 to 870)	290 (100 to 610)	660 (250 to 1,200)
Stroke medical costs to SUS	140 (54 to 300)	290 (100 to 640)	210 (75 to 450)	490 (190 to 880)
Stroke informal care costs	50 (19 to 100)	100 (36 to 220)	74 (26 to 160)	170 (65 to 310)
Total CVD-related costs	790 (320 to 1,400)	1,700 (720 to 3,000)	1,200 (490 to 2,200)	2,200 (1,100 to 3,900)
CVD medical costs to SUS	600 (240 to 1,100)	1,300 (540 to 2,300)	880 (370 to 1,600)	1,700 (790 to 3,000)
CVD informal care costs	190 (79 to 350)	400 (170 to 730)	280 (120 to 520)	550 (260 to 940)

All other counterfactual policy scenarios have a larger impact on healthcare savings than the current voluntary targets. From a public healthcare perspective, the mandatory implementation of the lowest global salt targets would save approximately Int$ 1.7 billion (95% UI: 0.7 to 3.0 billion), the front-of-pack warning labelling and voluntary limits would save around Int$ 1.2 billion (95% UI: 0.5 to 2.2 billion), and the universal use of 10% potassium table salt with the voluntary limits would generate savings of about Int$ 2.2 billion (95% UI: 1.1 to 3.9 billion).

## Discussion

We have identified potentially large future health and economic benefits if Brazil implemented more effective salt-reduction policies than continuing the current voluntary sodium targets. Our analysis suggests that additionally implementing stricter mandatory limits to sodium in processed and ultra-processed foods, front-of-pack nutritional labelling warnings for the excessive sodium content of foods and universal use of table salt with 10% potassium could substantially decrease the CVD burden and offer considerable cost savings to the public healthcare system and individuals compared to the continuation of the current voluntary salt-reduction targets in the country since 2019.

In Brazil, the population’s mean salt intake remains well above international recommendations, and almost 30,000 preventable CHD and stroke deaths annually are linked to excessive salt intake^5^. Reducing salt intake across multiple dietary sources and shifting dietary habits can be achieved only through the implementation of proven strategies. Relying solely on voluntary measures will yield limited health and economic benefits compared to other policies, and the costs of inaction, given the need for these additional strategies, are substantial. .Multicomponent salt reduction strategies using upstream and downstream interventions achieved considerable reductions in salt consumption at the population level, especially in countries such as Finland, Japan, Turkey and the UK. In a hierarchy of most effective strategies, mandatory food reformulation had the greatest impact on salt reduction, followed by voluntary reformulation, school interventions, short-term dietary advice, nutrition labelling and tax, community-based counselling, and health education media campaigns. In general, collective interventions on the food environment are more effective than those focused on changes in individual behaviours^13^.

Among the policy interventions we modelled, the WHO considers food reformulation for reducing the salt content of processed and ultra-processed foods as a best-buy strategy^37^. Recently updated regional limits set by PAHO^38^ and the global benchmarks for salt in these foods proposed by the WHO^39^ demonstrate that salt reduction is achievable in most food categories and that more stringent reductions are technically achievable based on existing country experiences. Future modelling studies must incorporate these new references to improve the impact of food reformulation by further reducing salt and other critical nutrients, yet consumption of ultra-processed foods may also impact cardiovascular outcomes through other mechanisms^40^.

Additionally, the impact of mandatory targets for salt in foods is greater than that of voluntary targets because they apply to all foods on the market and have stronger enforcement mechanisms^41^. The estimated reduction in daily salt intake in Brazil through voluntary targets has been modest compared with some other countries. However, even in the United Kingdom, considered a successful experience in voluntary food reformulation, more recently, the lack of robust and independent target-setting, monitoring and enforcement has undermined the salt reduction and its public health benefits^42^. As expected, implementing mandatory targets for more categories and using more restrictive limits on food salt content would have a larger impact on preventable CVD cases and deaths, savings to the National Health System, and informal health costs.

It is important that sodium targets also consider that a gradual reduction in the sodium content of foods may allow industries to develop new technologies to reduce or replace salt, thereby avoiding noticeable changes in the food taste^43,44^. Also, larger reductions during a short period may trigger rejection or compensatory behaviour by consumers^45^, while gradual reductions are unlikely to result in adding more table salt to foods or while cooking as compensatory behaviours^46,47^.

Front-of-pack nutritional labelling (FOPL) is an important tool for providing simplified nutritional information to consumers and informing them about products that can harm health as well as those that support healthier food choices. FOPL models are based on informing consumers about the critical nutrients in foods (sodium, sugars and fats) or a combination of favourable and unfavourable nutritional components through design systems using endorsement (healthy options), nutrient-specific (guideline daily amount systems, traffic-light and warning symbols) or summary schemes (healthy labels, health score or rating systems)^48,49^.

In Latin America, FOPL warning systems based on black octagons informing the excessive content of sodium, added sugars, and saturated and trans fats have been implemented successfully in several countries, including Chile, Uruguay, Argentina and Mexico^50–53^, many of which use the more restrictive nutritional profile systems developed by PAHO^20^. In Brazil, an alternative warning-label system was approved, proposing magnifying-glass symbols to indicate excess of sodium, added sugars and saturated fats, based in a national nutritional profile^54^. Nevertheless, previous studies in the country demonstrated that warning systems based on other symbols (triangles or octagons) and stricter nutrient profile systems are more effective in informing consumers and promoting healthier food choices^19,55^. Therefore, we modelled the impact of FOPL on excessive sodium in foods using octagon warnings and the nutrient thresholds recommended by the PAHO, although the expected impact on CVD and other disease outcomes might be much larger because of other nutrients not modelled in this study.

The final scenario, the universal use of 10% potassium-enriched table salt, was designed to model the impact of reducing sodium and increasing potassium intake, which will bring added benefits to cardiovascular health. The use of salt substitutes to lower sodium intake from table salt can be especially promising in regions where most of the salt consumed is added during home cooking or at the table^56^. In Finland, Pansalt has been used as a salt substitute since the 1970s, with 28% potassium chloride and 12% magnesium sulfate, and it is part of a comprehensive national salt reduction policy that contributed to reducing population salt intake by 40% over three decades^57^. More recently, large randomised control trials in China have tested salt substitutes (25% potassium) and showed significant reductions in sodium intake and blood pressure as intermediate results^58^ and, after five years, reduced the risk of major cardiovascular events by 13% and the risk of death by 12%^59^.

However, there is a growing concern about increasing potassium intake in settings with suboptimal monitoring of kidney and other conditions associated with hyperkalemia, which may lead to elevated potassium levels in the blood, despite the proven benefits for cardiovascular outcomes. On the other hand, the Chinese trial assessed hyperkalemia as a safety outcome and reported that the rate of serious adverse events attributed to hyperkalemia was not significantly higher in the salt-substitute group than in the regular-salt group. Additionally, modelling studies have estimated the impacts of potassium-enriched table salt on CVD and the risk of kidney disease in China and India and concluded that the cardiovascular health benefits overweigh potential hyperkalemia risks even among individuals with chronic kidney disease^60,61^. Consequently, the adoption of potassium-containing salt-substitutes should be carefully planned, monitored, and evaluated to minimise risks.

Even with the use of table salt with less sodium, strategies to modify population behaviour concerning discretionary (non-industrial) salt use must be strengthened, including the recommendations of the Brazilian Dietary Guidelines, which include consuming mostly unprocessed and minimally processed foods, using only small quantities of culinary ingredients like salt and avoiding ultra-processed foods^7^.

Within strategies based on sodium reduction targets, there is an effectiveness hierarchy: mandatory targets are superior to voluntary targets, and greater impacts are achieved through more stringent limits on sodium in foods^13^. If the current voluntary targets were made mandatory, almost 70% more CVD cases and deaths would be prevented, and adopting mandatory targets considering the first PAHO regional targets would more than double the health impacts compared to the voluntary targets. Additionally, in terms of front-of-pack nutritional labelling strategies, warnings are superior to traffic light systems, and regulatory approaches are more effective, so mandatory warnings can save 46% more CVD deaths and cases than voluntary traffic light systems (see S1 Appendix).

Different population groups may be more affected by certain policies than others, especially because of differences in dietary salt sources; therefore, adopting a comprehensive multistrategy approach is more impactful than fragmented strategies. For example, table salt is the main dietary source of salt among low-income families^6^, so salt substitutes may affect their consumption more significantly. Meanwhile, consumption of ultra-processed foods increases with income, so these individuals will substantially reduce their salt intake through front-of-pack warning labels and sodium-reduction targets.

This study provides policymakers and health advocacy groups with more accurate and timely real-world estimates of the likely effects of different salt-reduction strategies in Brazil compared to the continuation of the current voluntary targets. The overall impacts are likely to be an underestimation of the joint impact of mandatory and more stringent salt reduction targets for processed and ultra-processed foods, the implementation of FOPL warnings and the universal use of 10% potassium table salt, which together could reduce average per capita salt more significantly than isolated policies. However, additional strategies are needed to further reduce salt intake by improving the food environment to support healthier food choices implementing regulatory and fiscal policies and strengthening consumer education^13^.

### Strengths and limitations of this study

Among the strengths of this study, the IMPACT_NCD_ microsimulation framework has been extensively validated and replicated in different countries^11,62,63^ and the application of an IMPACT family microsimulation model in Latin America reinforces the use of modelling studies to inform policy in the region. Other strengths of this study include the use of representative population survey data and appropriate effect sizes from meta-analyses.

This study has potential limitations. First, salt consumption data were obtained from household purchases and adjusted by the total dietary calories, demonstrating that salt intake has steadily increased in Brazil, mostly because of increased consumption of processed and ultra-processed foods, while the consumption of table salt decreased^6^. Food records have shown different trends; nevertheless, they tend to underestimate the overall salt intake and especially table salt, which remains the main dietary source of salt in Brazil^64^. The salt density of the diet (g per kcal) estimated through food records is lower than that from household purchases, although both estimates show that excessive salt intake is a relevant public health issue. Therefore, it is paramount that dietary surveys include more accurate estimates of table salt consumption and that new health surveys include 24-h urinary sodium excretion estimates to provide more accurate future trends in salt/sodium intake, which can be incorporated into modelling studies.

Second, the effect estimates in the model are based on interventional and prospective observational studies and may, therefore, be subject to bias and confounding. However, our estimates may be conservative and underestimate the full health and economic benefits of all salt reduction strategies that were analysed: (1) the model only evaluated diseases mediated through BP, although decreasing salt consumption could have beneficial effects upon other health burdens such as gastric cancer^65^; (2) the model assumed a median duration of 5 years from changes in SBP to CVD risk and only considered the first cardiovascular episode, therefore, only primary prevention benefits were quantified; (3) food reformulation by industries might also increase potassium intake through substitution of NaCl with KCl^66^, thereby providing potential benefits that were not included in the model; (4) SBP response to sodium reduction tends to be greater among individuals of African descent, and Brazil has a large proportion of Black and mixed-race (pardo) populations whose specific dose–response was not explicitly modelled. Third, local studies based on real-world simulations of food purchases comparing the two front-of-pack labelling schemes and their nutrient profiles were not available at the time of the analyses, so we based our comparison on results from an online survey, which may overestimate the proportion of changes in food purchasing intention^67^. Fourth, informal costs were based on evidence from European countries. As a result, our estimates might be conservative as they do not include Brazil’s private care costs and medical costs from the private sector, which covers 30% of the population. Finally, the model relies on several assumptions regarding industry compliance with regulations and the time-lags between policy onset and changes in food composition and population salt intake, and these assumptions influence the estimates throughout the modelling process.

## Conclusions

Our findings suggest that making the national salt reduction targets for processed and ultra-processed foods in Brazil mandatory, and adopting more stringent limits, could generate substantial health benefits for the population and cost savings for the National Health System and informal health care costs. Additional health and economic impacts can be achieved by implementing other policies, such as front-of-pack warnings on excessive salt in these foods and the universal use of 10% potassium table salt. However, even with these strategies, salt intake will remain high in the country, so additional policies focused on all dietary sources of salt must also be implemented, aligned with the food-based dietary guidelines and comprehensive approaches to healthy diets.

## Supplementary Information

Supplementary Information.

## Data Availability

All relevant data are within the paper, supporting information files, and the GitHub repository (https://github.com/ChristK/IMPACTncd_Br/tree/voluntary_reformulation). The source code of the model is available at https://github.com/ChristK/IMPACTncd_Br/tree/voluntary_reformulation. All data inputs are available as .csv files in this repository, except the Population, Household Budget and Health Survey for Brazil microdata, which are publicly accessible through the Brazilian Institute of Geography and Statistics ([https://www.ibge.gov.br/], (https:/www.ibge.gov.br)), and the hospitalisation and mortality data, which is also publicly available through the Brazilian National Health System’s Department of Informatics (http://tabnet.datasus.gov.br/).

## Abbreviations

CHD: Coronary heart disease
NCD: Non-communicable disease
CVD: Cardiovascular disease
DALYs: Disability-adjusted life-years
SBP: Systolic blood pressure
BP: Blood pressure
SUS: Brazilian national health system
PNS: National health survey
POF: Household budget survey
SIH-SUS: Hospital information system
SIM: Mortality information system
UI: Uncertainty interval
WHO: World health organization
