# Supplementary material for: Estimated health and economic effects of different salt reduction strategies on cardiovascular disease in Brazil: a microsimulation analysis
Source: Sci Rep. 2026 Apr 30;16:20179. doi: 10.1038/s41598-026-49991-1 (PMC13324168; doi:10.1038/s41598-026-49991-1)
Supplement: Supplementary file 1 — Supplementary Information. [file 41598_2026_49991_MOESM1_ESM.pdf]

Additional file 1

Supplementary Technical Appendix

The authors have provided this Appendix to give readers additional information about their work.

Supplement to: Estimated the health and economic effects of different salt reduction strategies on cardiovascular disease in Brazil: a microsimulation analysis.

Eduardo Augusto Fernandes Nilson, Jonathan Pearson-Stuttard, Brendan Collins, Maria Guzman-Castillo, Simon Capewell, Martin O'Flaherty, Chris Kypridemos [4]

This Technical Appendix was adapted from a previously published one in:

Nilson, E.A.F., Pearson-Stuttard, J., Collins, B. et al. Estimating the health and economic effects of the voluntary sodium reduction targets in Brazil: microsimulation analysis. BMC Med 19, 225 (2021). https://doi.org/10.1186/s12916-021-02099-x.

Appendix 1: CHEERS 2022 Checklist

CHEERS 2022 Checklist

Topic	No.	Item	Location where item is reported
**Title**			
	1	Identify the study as an economic evaluation and specify the interventions being compared.	Title/Page 1
**Abstract**			
	2	Provide a structured summary that highlights context, key methods, results, and alternative analyses.	Abstract/Page 2
**Introduction**			
**Background and objectives**	3	Give the context for the study, the study question, and its practical relevance for decision making in policy or practice.	Introduction/Pages 3-5
**Methods**			
**Health economic analysis plan**	4	Indicate whether a health economic analysis plan was developed and where available.	Appendix
**Study population**	5	Describe characteristics of the study population (such as age range, demographics, socioeconomic, or clinical characteristics).	Methods/Page 6
**Setting and location**	6	Provide relevant contextual information that may influence findings.	Methods/Page 6
**Comparators**	7	Describe the interventions or strategies being compared and why chosen.	Methods/Page 5
**Perspective**	8	State the perspective(s) adopted by the study and why chosen.	Methods/Page 5
**Time horizon**	9	State the time horizon for the study and why appropriate.	Methods/Pages 8
**Discount rate**	10	Report the discount rate(s) and reason chosen.	Methods/Page 12
**Selection of outcomes**	11	Describe what outcomes were used as the measure(s) of benefit(s) and harm(s).	Methods/Pages11-12

Topic	No.	Item	Location where item is reported
**Measurement of outcomes**	12	Describe how outcomes used to capture benefit(s) and harm(s) were measured.	Methods/Pages 11-12
**Valuation of outcomes**	13	Describe the population and methods used to measure and value outcomes.	Methods/Page 6
**Measurement and valuation of resources and costs**	14	Describe how costs were valued.	Methods/Pages 12
**Currency, price date, and conversion**	15	Report the dates of the estimated resource quantities and unit costs, plus the currency and year of conversion.	Methods/Page 12
**Rationale and description of model**	16	If modelling is used, describe in detail and why used. Report if the model is publicly available and where it can be accessed.	Methods/Pages 6-9 and Appendix
**Analytics and assumptions**	17	Describe any methods for analysing or statistically transforming data, any extrapolation methods, and approaches for validating any model used.	Table 1 and Appendix
**Characterising heterogeneity**	18	Describe any methods used for estimating how the results of the study vary for subgroups.	Methods/Page 6
**Characterising distributional effects**	19	Describe how impacts are distributed across different individuals or adjustments made to reflect priority populations.	Methods/Page 6
**Characterising uncertainty**	20	Describe methods to characterise any sources of uncertainty in the analysis.	Methods/Pages 12
**Approach to engagement with patients and others affected by the study**	21	Describe any approaches to engage patients or service recipients, the general public, communities, or stakeholders (such as clinicians or payers) in the design of the study.	Not applicable
**Results**			
**Study parameters**	22	Report all analytic inputs (such as values, ranges, references) including uncertainty or distributional assumptions.	Table 1
**Summary of main results**	23	Report the mean values for the main categories of costs and outcomes of interest and summarise them in the most appropriate overall measure.	Tables 2 and 3

Topic	No.	Item	Location where item is reported
**Effect of uncertainty**	24	Describe how uncertainty about analytic judgments, inputs, or projections affect findings. Report the effect of choice of discount rate and time horizon, if applicable.	Tables 1 and 2
**Effect of engagement with patients and others affected by the study**	25	Report on any difference patient/service recipient, general public, community, or stakeholder involvement made to the approach or findings of the study	Appendix
**Discussion**			
**Study findings, limitations, generalisability, and current knowledge**	26	Report key findings, limitations, ethical or equity considerations not captured, and how these could affect patients, policy, or practice.	Discussion, Page 17
**Other relevant information**			
**Source of funding**	27	Describe how the study was funded and any role of the funder in the identification, design, conduct, and reporting of the analysis	Page 24
**Conflicts of interest**	28	Report authors conflicts of interest according to journal or International Committee of Medical Journal Editors requirements.	Page 25

From: Husereau D, Drummond M, Augustovski F, et al. Consolidated Health Economic Evaluation Reporting Standards 2022 (CHEERS 2022) Explanation and Elaboration: A Report of the ISPOR CHEERS II Good Practices Task Force. Value Health 2022;25. doi:10.1016/j.jval.2021.10.008

A high-level description of the IMPACT$_{NCD-BR}$ model

The IMPACT$_{NCD-BR}$ model is a discrete-time dynamic stochastic microsimulation model. It is an implementation of the IMPACT $_{NCD}$ modelling framework that has been used previously to model the impact of sodium reduction policies in England and the US (1)(2)(3)(4).

Within the IMPACT$_{NCD-BR}$ model, each unit is a synthetic individual and is represented by a record containing a unique identifier and a set of associated attributes. For this study, we considered age, sex, sodium consumption (considering industrial and non-industrial sources of dietary sodium, i.e. the sodium added by industries to foods and the other sources of sodium in the diet, including table salt), and SBP. A set of stochastic rules is then applied to these individuals, such as the probability of developing coronary heart disease (CHD) or dying, as the simulation advances in discrete annual steps. The output is an estimate of the burden of CHD and stroke, in the synthetic population, including both total aggregate change and, more importantly, the distributional nature of the change (Figure 1).

The IMPACT$_{NCD-BR}$ model is a complex model that simulates the life course of synthetic individuals and consists of four simulation engines: the 'population' engine, the 'disease' engine, the 'health economics' engine, and the 'policy' engine. The description of the processes in each of the engines is fully described in the following chapters. The description focuses on the model's rationale from an epidemiological perspective and complements the source code available at Github (https://github.com/ChristK/IMPACTncd_Br/tree/voluntary_reformulation) covered by the GPL v3 licence. Figure S1 depicts the model's logic, and Tables A and B summarise the sources of the input parameters and the main assumptions and limitations, respectively.

Technical information

The IMPACT$_{NCD-BR}$ model is being developed in R v3.6.1. The IMPACT$_{NCD-BR}$ model is built around the R package 'data.table', which imports a new heavily optimised data structure in R. Most functions that operate on a data table have been coded in C to improve performance. In addition, to ensure statistical independence of the pseudo-random number generators running in parallel, the R package 'doRNG' was used to produce independent random streams of numbers generated by L'Ecuyer's combined multiple-recursive generator .

Figure 1. A logical framework for the IMPACT$_{NCD-BR}$ model

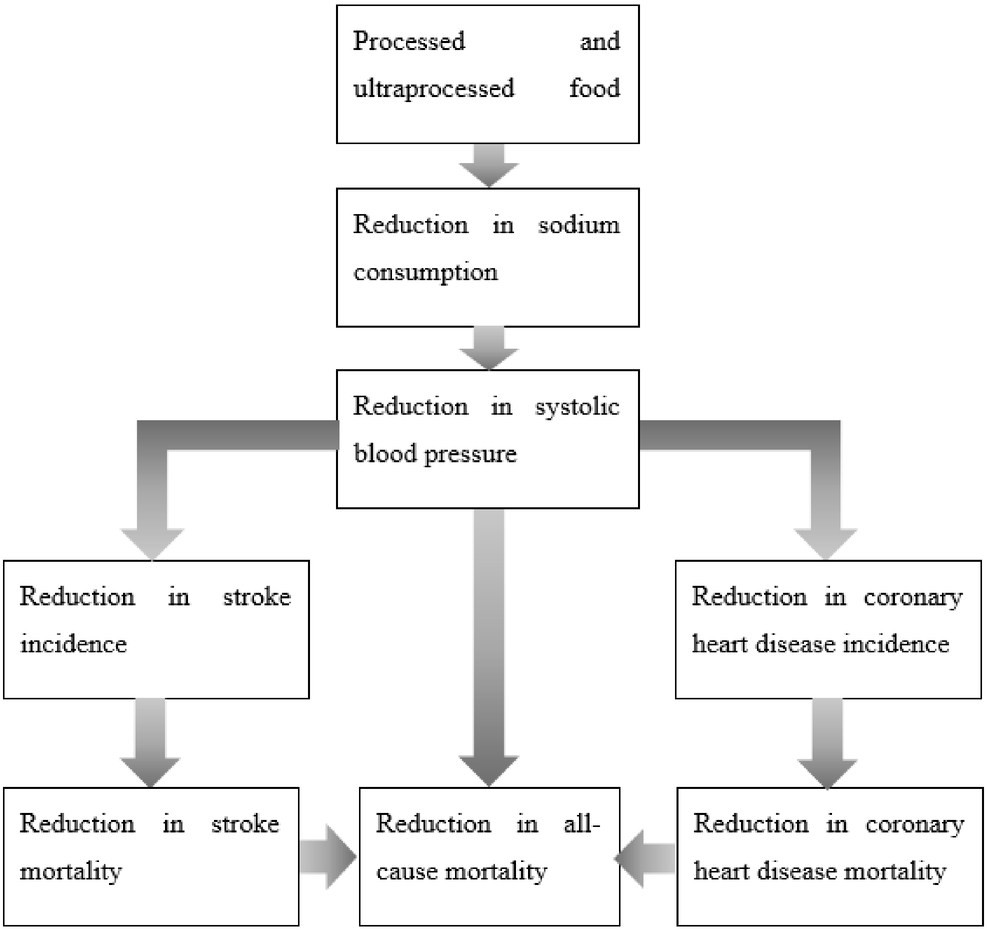

Population engine

The population module includes the demographic module and the exposure module.

Demographic module

Synthetic individuals enter the simulation in the initial simulation year (2019 for this study). The number of synthetic individuals that enter the simulation is user-defined, and for this study was set to 400,000. First, the algorithm ensures that the sample's joint age and sex distribution is like the Brazilian population in 2013 (5). Then it creates backward and forward projections of the synthetic population that are essential to model exposure time trends and time lags between exposures and diseases.

The backward projection of the synthetic population goes back to 2003; therefore, the maximum time lag we allow in the model is ten years. As everyone alive and older than 30 years old in 2018 was alive in 2003, the algorithm creates the back projections by appropriately reducing the synthetic individuals' age while keeping constant all other variables.

For the forward projections, we project until the year 2038, and the algorithm increases the age of the synthetic individuals while keeping the sex variable constant. For forward projections, mortality needs to be considered. We describe mortality with the disease module as disease-specific mortality, which is closely related to disease prevalence. The model follows an open cohort approach. Every simulated year from 2019 onwards, a new cohort of 30 years old synthetic individuals enters the model. The same sources inform the size of the cohort and the joint age and sex distribution we described above. For example, in 2020, the new 30-year old cohort will be informed by the population size and the joint age and sex distribution of 29 years old in 2019. The approach may be crude; however, the final model outputs are directly standardised to Brazilian population projection (5) to minimise the bias.

Exposure module

This module simulates the adult life course sodium and SBP exposures of the synthetic individuals based on POF 2008-2009[1] the National Health Survey 2013 (PNS 2013) (6) (7). For all simulated

[1] We used the indirect estimations of sodium intake from the Household Budget Survey (POF 2008-2009) for two reasons: 1) they were closest to the baseline of target setting (2010) and the model framework was designed before the publishing of spot urine analyses from the 2013 National Health Survey; 2) Most importantly, the POF 2008-2009 provided more detailed age and sex distributions and the identification of food categories and their sodium

exposures, we followed the same general principles. First, we fitted a Generalised Additive Model for Location, Scale and Shape (GAMLSS)[2] to the data with the exposure of interest as the dependent variable and some functions of age and sex as independent variables (8). Then, we use the GAMLSS model to predict the exposure level of every synthetic individual in the simulation and simulate individualised risk factor trajectories for all synthetic individuals, based on their age and sex, that were estimated from the sociodemographic module.

The above approach provides us with equations to estimate the distribution of an exposure for a given age and sex. When the synthetic individual enters the simulation, a set of random numbers between 0 and 1 and size equal to the number of the modelled exposures is allocated to her. Each one of them represents the percentile of the relevant exposure distribution. The principle is that synthetic individuals retain their percentiles throughout their life course (this is known as the rank stability assumption). For example, in 2019, a 40-year-old male synthetic individual with SBP of 120 mmHg has an SBP percentile of 0.52. Twenty years later, the same synthetic individual has retained his percentile score for SBP. However, his SBP is now estimated to 137.6 mmHg because the SBP distribution has changed to reflect the SBP of 60-year-old men.

Finally, to model the exposure to sodium intake, we fitted to GAMLSS models, one for industrial and one for non-industrial sources of dietary sodium. Because these two exposures were correlated, we simulated their linear correlation by using correlated random number streams for the percentiles.

---

content and total consumption.

[2] These are flexible statistical models that can make all parameters of an assumed distribution for the dependent variable, conditional to some function of the independent variables. For example, GAMSS can model both the mean and the standard deviation of a dependent normally distributed variable, while a linear regression only models the mean.

Disease engine

The previous two modules for demographics and exposure generate a dynamic close-to-reality synthetic population composed of each synthetic individual's adult life course exposures. The disease module then translates these exposures to disease incidence, using a population attributable risk approach (PAF). Thus, we will first describe how disease incidence is simulated in the model and then how the model simulates mortality.

Disease incidence

To estimate the individualised annual probability of a synthetic individual developing a specific disease conditional on their risk exposures, we follow a 3-step approach:

Step 1. The proportion of incidence attributable to SBP by age and sex is estimated, assuming a mean time lag of 5 years between exposure and disease, reflecting the best possible empirical data based on the observation period of cohort studies and time to risk reversal in randomised clinical trials (9)(10).[3] The time lag varies stochastically between 2 and 10 years following a shifted binomial distribution in each iteration.

Step 2. The portion of the disease incidence attributable to SBP is estimated and subtracted from the total incidence for 2019.

Step 3. The probability of developing the disease is estimated for each individual in the synthetic population and is used in an independent Bernoulli trial to select those who finally develop the disease.

The implementation of the above method is described in more detail using CHD as an example. The exact process is used for stroke outcomes.

Step 1

PAF is an epidemiological measure that estimates the proportion of the disease attributable to an associated risk factor. It depends on the relative risk associated with the risk factor and the prevalence of the risk factor in the population. In a microsimulation context where exposure to risk

---

[3] We assumed no lag time between a change in sodium intake and impact on SBP, as this happens within few weeks (31).

factors are known at the individual level, PAF can be calculated using the formula:

$$PAF = 1 - \frac{n}{\sum_{i=1}^{n} RR_i}$$

where n is the number of synthetic individuals in the population, and RR_i are the relative risks of the SBP associated with CHD for each individual i. We calculated PAF based on the above formula stratified by age and sex only in the initial year of the simulation. Consistent with findings from the respective meta-analyses used for the $\text{IMPACT}_{\text{NCD-BR}}$ model, SBP below 110 mmHg, was considered to have a relative risk of 1 . All the relative risks were taken from published meta-analyses.

Step 2

The incidence of CHD not attributable to the modelled risk factors can be estimated by the formula:

$$I_{Theoretical\,minimum} = I_{Observed} * (1 - PAF)$$

Where $I_{Observed}$ is the CHD incidence and PAF is from Step 1. $I_{Theoretical\,minimum}$ represents CHD incidence if SBP was at optimal levels across the population. To account for future time trend in CHD incidence that is not attributable to SBP, the model updates $I_{Observed}$ every simulated year. For this, we assume that half of the forecasted annual change in CHD mortality is attributed to changes in CHD incidence and the other half to changes in CHD case fatality. We based this assumption on observational evidence from England and modelling studies in England and the US (1)(2)(3)(4). Furthermore, we included this assumption in our probabilistic sensitivity analysis.

Stage 3

Assuming that $I_{Theoretical\,minimum}$ is the annual baseline probability of a synthetic individual to develop CHD for a given age and sex due to risk factors not included in the model, the individualised annual probability of developing CHD, $\mathbb{P}(\text{CHD} \mid \text{age, sex, exposures})$, given his/her risk factors were estimated by the formula:

$$\mathbb{P}(CHD \mid age, sex, exposures) = I_{Theoretical\,minimum} * RR_i$$

Where RR_i the relative risk that is related to the SBP of the synthetic individual, same as in stage 1.

The method described above can be used only when the disease incidence in the population is known. However, the true incidence of CHD (and stroke) is largely unknown. While several estimates exist, all have limitations. Therefore, for the estimation of CHD incidence by age and

sex, we opted for a modelling solution to synthesise all the available sources of information and minimise bias. Specifically, we used the Information System on Mortality (*Sistema de Informações de Mortalidade* - SIM) database (19) to extract mortality rates for CHD (ICD-10: I20–I25) for the years 1999–2015, stratified by age and sex. We also estimated CHD's self-reported prevalence by age and sex from the PNS 2013 (59). We used both prevalence and mortality rates to inform the World Health Organisation (WHO) DISMOD II model (11). DISMOD II is a multi-state life table model that can estimate the incidence, prevalence, mortality, fatality, and remission of a disease when information about at least three of these indicators is available. A similar approach has been followed by the Global Burden of Disease team and other groups (12)(13). We considered CHD an incurable chronic disease (i.e. remission rate was set to 0); therefore, the derived DISMOD II incidence refers to the first-ever manifestation of angina or AMI excluding any recurrent episodes. For the DISMOD II calculations, we assumed that incidence and case-fatality had each been declining by 2% (relative) over the last 20 years. The derived CHD incidence and prevalence rates were used as an input for $\text{IMPACT}_{\text{NCD-BR}}$. A similar approach was used for stroke.

For the initial simulation year, some synthetic individuals need to be allocated as prevalent cases for each of the modelled diseases. Therefore, we use DISMOD II prevalence estimates to identify prevalent disease cases by age, sex.

Mortality

All synthetic individuals are exposed to the risk of dying from any of their acquired modelled diseases or any other non-modelled cause in a competing risk framework. The $\text{IMPACT}_{\text{NCD-BR}}$ model is calibrated to observed CHD, stroke, and any-other-cause mortality for 2019 and mortality forecasts for the years 2019–2038. For years after 2017, coherent functional demographic models by sex and age were fitted to the reported CHD, stroke, and any-other-cause mortality rates from 2000 to 2017 (14) and then were projected to the simulation horizon using the R package 'demography' (15). Functional demographic models are generalisations of the Lee-Carter demographic model, influenced by ideas from functional data analysis and non-parametric smoothing (16). The coherent approach ensures that subgroup forecasts do not diverge over time. Finally, we used the observed and forecasted mortality rates to create life tables for each simulated year by age, sex, and disease (CHD, stroke, any-other-cause). We applied the any-other-cause life tables to all synthetic individuals and the CHD and stroke life tables to prevalent cases of CHD and stroke only, respectively. For the synthetic individual that died of more than one causes in a specific year, a cause was randomly selected to minimise bias.

In reality, hypertensive individuals have a higher risk of dying not only of CHD and stroke but also from a spectrum of other diseases. To account for this and minimise bias, the $\text{IMPACT}_{\text{NCD-BR}}$ model inflates the any-other-cause mortality rates for hypertensive synthetic individuals in the model (Figure A link between SBP and all-cause mortality) while it deflates it for non-hypertensives. The algorithm ensures that the total number of hypertensive and non-hypertensive synthetic individuals who die every year from any-other-cause is equal to the defined one in the life table. The algorithm is based on the PAF approach, and the relative risk was derived from an individual level meta-analysis by Stringhini *et al.* (17). In this meta-analysis, the relative risk of all-cause mortality for hypertensives was 1.31 (1.24–1.38), and the relative risk of non-CVD-non-cancer mortality was 1.29 (1.21–1.38). Hence, we used a relative risk of 1.3 in the $\text{IMPACT}_{\text{NCD-BR}}$ model.

Health economics engine

In the previous two modules, the IMPACT$_{NCD-BR}$ model creates synthetic individuals with traits similar to those observed in the Brazilian population and tracks their future exposures to sodium and SBP and important events (first manifestation of CHD and stroke, death from CHD, stroke, or any other cause).

Disease costs

The IMPACT$_{NCD-BR}$ model applies CHD, stroke, and hypertension costs to these diseases' cases during the simulation. These costs are mean estimates by age and sex.

Disease costs per person-year were derived from the National Health System's Hospital Information System (SIH/SUS) (18). We assumed constant medical costs in US dollars. Medical costs per person-year for CHD and stroke were calculated by dividing total hospitalisation costs by the number of people with each condition in 2019.

Informal care costs for CHD and stroke were based on the ratio of hospital care to other medical costs in Europe from a study by Leal *et al.* (19). We assumed no informal care costs for hypertension alone.

Policy engine

Until now, the description of the $IMPACT_{NCD-BR}$ model was for the baseline scenario. The policy module translates the policy scenarios to be modelled by the $IMPACT_{NCD-BR}$ model. Changes in sodium consumption are translated into changes in SBP using the meta-regression equation by Mozaffarian *et al*. (20) by age and hypertensive status.[4] The new SBP is used in the disease module, and updated CHD and stroke risks are calculated for every synthetic individual, with new outcomes. Therefore, new life courses for all synthetic individuals are simulated as a result of the modelled policies. At the end of the simulation, the model compares all alternative life courses with the baseline life course for each synthetic individual and calculates the outputs.

Policy scenarios

Two sodium target scenarios for processed and ultra-processed foods were modeled: the continuity of the current voluntary sodium targets and the implementation of mandatory targets based on the lowest targets implemented globally in 2019, using data from official national food labelling surveys in 2017 and 2019 to estimate changes in food composition. For the voluntary target scenario, we have considered changes in food composition from the most recent documented official monitoring (21), assuming that sodium content was reduced for the targeted food categories only by industries that have voluntarily committed to the national sodium targets (which correspond to a 70% market share in the country). For the mandatory sodium target scenario, we selected the lowest targets in force in 2019 for Brazil, England, Canada, the United States, Argentina, and South Africa and the first regional targets set by PAHO (22)(23)(24)(25)(26) and used these values to map the Brazilian food survey data, reestimating the sodium content of all targeted food categories in the market. We assumed that the reformulation of food products would adjust sodium content to targets in 2019 and that this would lead to an immediate change in sodium intake in synthetic individuals according to the level of reformulation. We also assumed that the reformulated products would, after that, sustain their sodium content.

---

[4] We apply this equation only to synthetic individuals with sodium consumption above the optimal level of sodium consumption. Hence, the sodium consumption projection of the baseline scenario is not directly used during this calculation. Only the change in sodium consumption is important and is translated in SBP and health outcomes change.

The regulatory front of package labelling (FOPL) warnings for excessive salt in foods modeled the impact of adopting octagon warnings with messages of "high sodium" in foods considering the thresholds recommended by the Pan-American Health Organization (27) and assuming that part of the population would replace foods with warnings with fresh and minimally processed foods according to Khandpur et al (28).

The salt substitute scenario assumed that all table salt in Brazil was replaced with a 10% potassium table salt, considering the impact added impact of sodium reduction and potassium increase. The impact of increasing potassium intake was estimated using a comparative risk assessment approach in parallel to the sodium model, considering the meta-analysis by D'Elia et al (29) and similar lag times to sodium for the impact on cases and deaths prevented or postponed.In 2010, the Brazilian Ministry of Health proposed a voluntary approach to reducing the average sodium content of the industrialised foods, which contributed to over 90% of the sodium intake from processed and ultra-processed foods, partnered by the Brazilian Association of Food Industries (Abia) (11). The targets were set as gradually decreasing upper limits to the salt content in foods in two-year steps. Because target setting was negotiated individually for each food category, the agreements (Terms of Commitment) were released gradually, from 2011 to 2013, until all priority food categories had defined targets. The targets were monitored through nutritional label surveys in 2013-2014 and 2017-2018, which analysed the compliment to the targets and the reduction in the average sodium content of foods during the period (21).

Separately, we linked the priority food categories to the POF 2008-2009 foods and codes to re-estimate sodium intake at baseline and 2017. Therefore, to model the effect of the proposed policy on the modelled population, we developed the algorithm below:

This approach allows incorporating sodium consumption time trends in the calculations and provides enough granularity of the policy effect (by age, sex, and sodium consumption) without being too computationally intensive. Nevertheless, it does not address potential behavioural changes of the population due to the reformulation and does not account for foods prepared in food outlets and restaurants.

Uncertainty and sensitivity analysis

The $\text{IMPACT}_{\text{NCD-BR}}$ model implements a 2nd order Monte Carlo approach to estimate uncertainty intervals (UI) for each scenario (30). Each simulation, which includes all policy scenarios, runs 2000 times. A different set of input parameters is used for each iteration by sampling from the

respective distributions[5] of input parameters, and a different sample of 400,000 synthetic individuals is drawn. Then, the life course of every synthetic individual is simulated for the baseline, and all policy scenarios and the outcomes are collected and summarised for the population annually.[6] Therefore, all model outputs (cases and deaths prevented or postponed) are separately estimated for each iteration and conditional on the set of model inputs.

The framework allows stochastic uncertainty, parameter uncertainty, and individual heterogeneity reflected in the reported UI. The following example illustrates the different types of uncertainty that were considered in the $\text{IMPACT}_{\text{NCD-BR}}$ model. Let us assume that the annual risk of CHD is 5%. If we apply this risk to all individuals and randomly draw from a Bernoulli distribution with p = 5% to select those who will manifest CHD, we only consider stochastic uncertainty. If we allow the annual risk for CHD to be conditional on individual characteristics (i.e. age, sex, exposure to risk factors), then individual heterogeneity is considered. Finally, when the uncertainty of the relative risks due to sampling errors is considered in estimating the annual risk for CHD, the parameter uncertainty is considered. From these three types of uncertainty, only the parameter uncertainty can be reduced from better studies in the future.

The structure of the model is grounded on fundamental epidemiological ideas and well-established causal pathways; therefore, we considered this type of uncertainty relatively small and did not study it. However, the discrete-time nature of the model can potentially introduce bias in cases where the synthetic individual dies more than once within a year, and the model cannot identify which event happened first. As we described earlier, to minimise this type of bias, we randomly select one of the events to be considered as it happened before all others, whenever these cases arise during the simulation.

Input uncertainty

The sources of uncertainty we considered were:

1. The sampling error of the baseline sodium intake.

---

[5] We assumed log-normal distributions for relative risks and hazard ratios, normal distributions for coefficients of linear regression equations, and PERT distributions for other parameters.

[6] For instance, if a synthetic individual developed CHD at the age of 50 in the baseline scenario and at the age of 60 in a policy scenario, this is counted as 10 CHD cases prevented or postponed, as a result of the policy.

2. The sampling error of the baseline SBP.
3. *The sampling error of the relative risks of SBP on CHD, stroke, and any-other-cause mortality*. We used the reported relative risks and their confidence intervals to construct log-normal (uniform for *any-other-cause mortality*) distributions.
4. The uncertainty around the lowest exposure to sodium below which no risk is observed. We used evidence in Mozaffarian et al. as parameters for a Pert distribution.
5. The uncertainty around the lowest exposure to SBP below which no risk is observed. We used evidence in Singh et al..
6. *The uncertainty around the effect of sodium on SBP*. We used the meta-regression equation in Mozaffarian *et al*. Each time the model uses the equation, a new set of coefficients was sampled from their respective normal distributions.
7. *The uncertainty around the lag time of SBP exposure and disease outcomes.* The distribution 1 + Binomial(9, (5-1)/9) to vary the lag time between 1 and 10 years (median 5 years).
8. *The uncertainty around the true incidence and prevalence rates of CHD and stroke*. We described in page 14 how we used DisMod II to estimate the incidence rate of CHD and stroke. We fitted beta distributions by age, sex, and race/ethnicity, assuming the 0.025 percentile to be half of the central estimate, the median the central estimate, and the 0.975 percentile double the central estimate.
9. *The uncertainty of mortality forecasts*. We incorporated the predictive uncertainty of the mortality forecasts into the $\text{IMPACT}_{\text{NCD-BR}}$ model estimates.
10. The uncertainty around the assumption that half of the forecasted annual change in CHD and stroke mortality is attributed to changes in CHD and stroke incidence, respectively. We allowed this assumption to vary, independently for each disease, between 0% and 100% following a uniform distribution.

Outputs

We summarise the output distributions of the $\text{IMPACT}_{\text{NCD-BR}}$ by reporting the medians and 95% uncertainty intervals (UI). We also plotted the annual probability that a scenario was cost-effective or cost-saving over the simulation period. Table J presents model estimates for the baseline

scenario.

Cases (Deaths) prevented or postponed by comparing the life course of each specific individual in the baseline scenario with its life course in the policy scenario.

All outputs can be stratified by year, age, sex, and disease. Moreover, outputs are scaled to the Brazilian population (from the 400,000 synthetic individuals sample).

It is important to not misinterpret 95% UIs as 95% confidence intervals (CI) and overlapping UIs as 'evidence against statistical significance.' This does not apply to our model outputs because the scenarios share common model inputs as explained above and should not be treated as 'independent' from a statistical perspective.

Internal validation

The following plots depict the internal validation of the model.

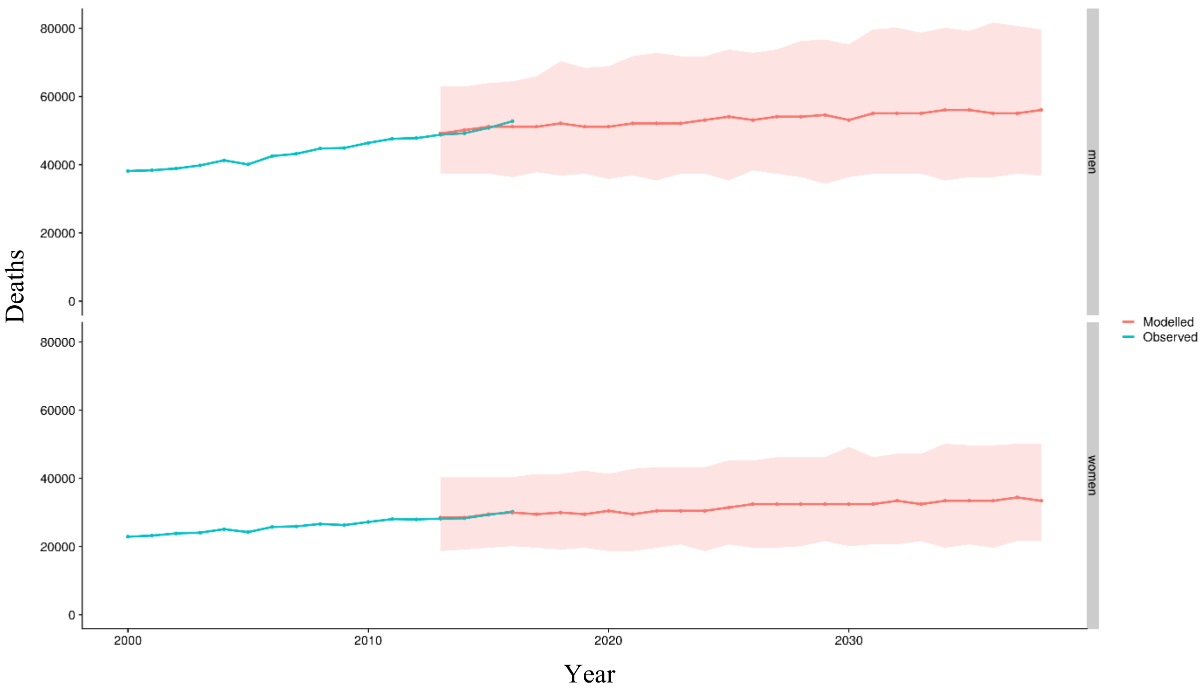

Figure 2. Coronary heart disease mortality by sex. Observed versus modelled number of deaths.

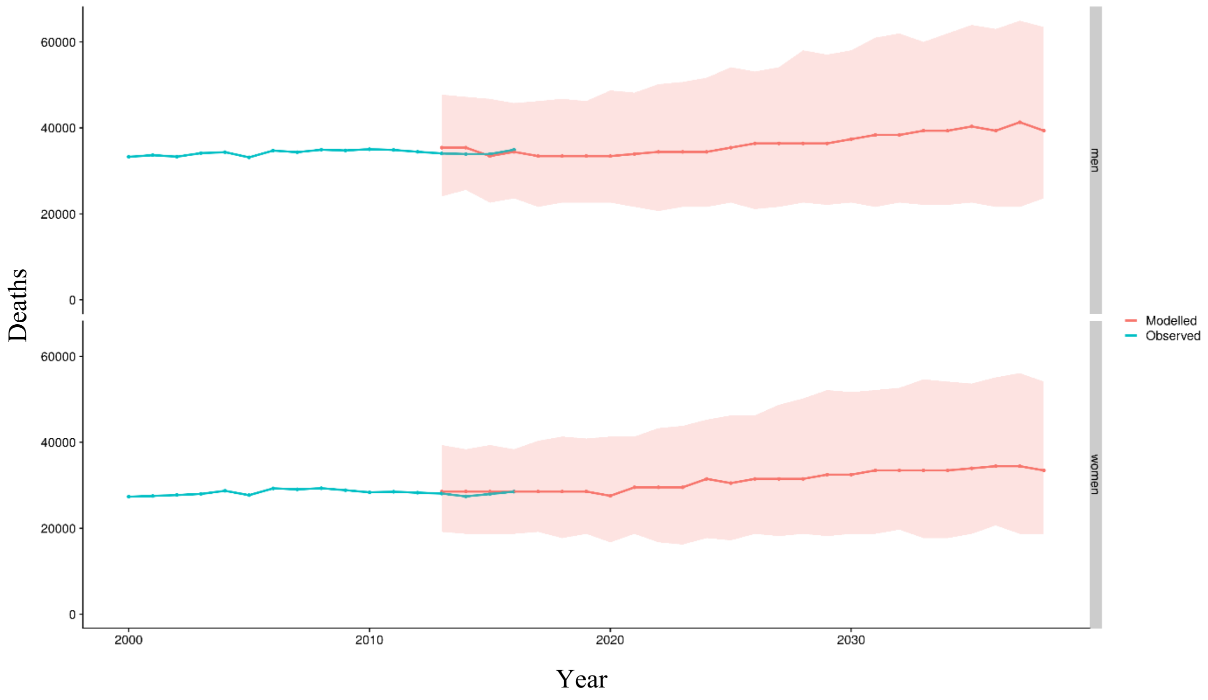

Year

Figure 3. Stroke mortality by sex. Observed versus [illegible] number of deaths.

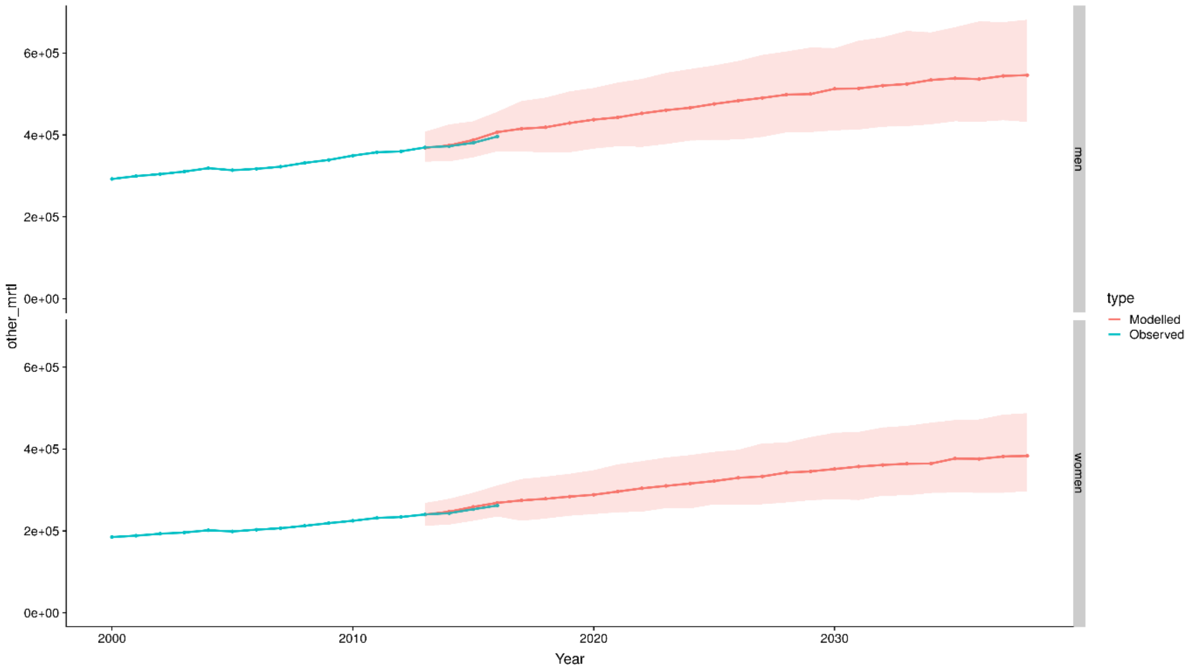

Figure 4. Other (non-coronary heart disease, non-stroke) mortality by sex. Observed versus modelled number of deaths.

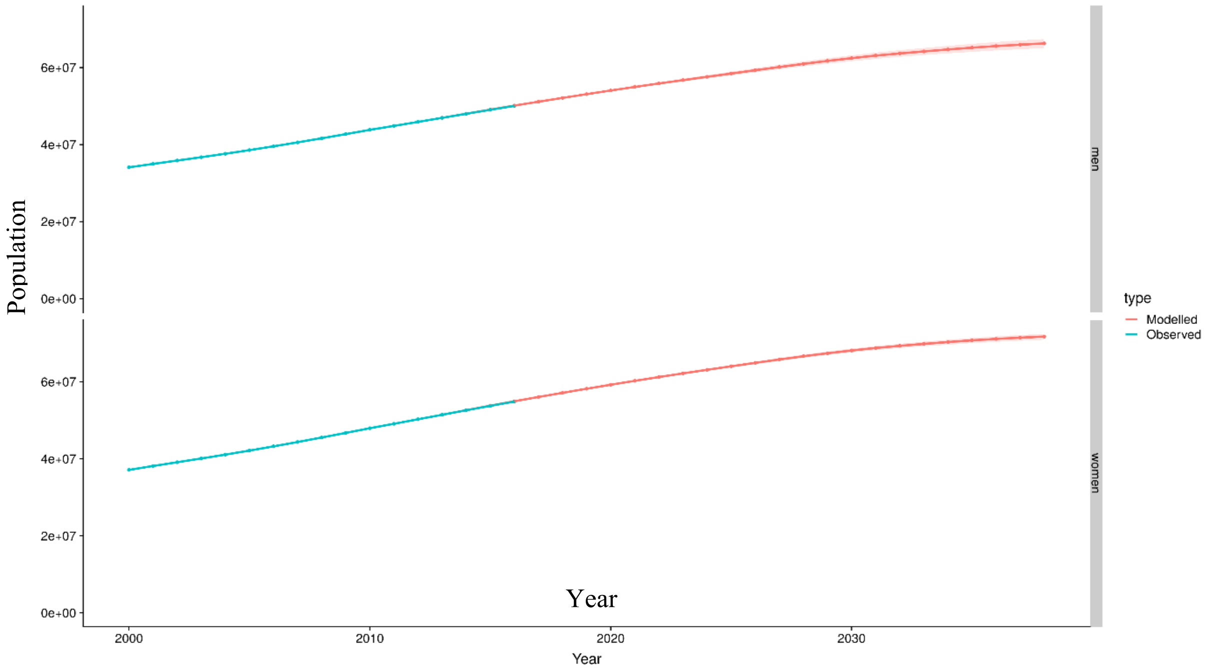

Figure 5. Population size by sex. Observed versus modelled number of deaths.

Tables

Table S1. The IMPACT$_{NCD-BR}$ model data sources.

Parameter	Outcome	Details	Comments	Source
Population size estimates	Population	Resident population from 2010 National Census and intercensal estimates	Stratified by year, age, and sex	Brazilian Institute of Geography and Statistics (IBGE) – online reports tables and microdata
Population projections	Population	2012–2060 Brazilian population projections produced by the Brazilian Institute of Geography and Statistics (IBGE)	Stratified by year, age, and sex	Brazilian Institute of Geography and Statistics (IBGE) – online reports and tables
Mortality	Deaths from CHD, stroke, and any other non-modelled causes	Underlying cause of death 2000-2017	Stratified by year, age, sex, and cause of death	Ministry of Health of Brazil. National Mortality Information System (*Sistema de Informações de Mortalidade* – SIM). Underlying cause of death 2000-2016 based on the ICD-10 codes. 2018

Exposure to sodium	Exposure of individuals	National Household Budgetary Surveys (POF)	Anonymized, individual-level data sets. Years 2008-2009.	Brazilian Institute of Geography and Statistics (IBGE) – POF 2008-2009 and 2017-2018 (*Pesquisa de Orçamentos Familiares*) online public microdata and reports
Exposure to systolic blood pressure	Exposure of individuals	National Health Survey (PNS)	Anonymized, individual-level data sets. Year 2013.	Brazilian Institute of Geography and Statistics (IBGE) – PNS 2013 (*Pesquisa Nacional de Saúde*) online public microdata and reports
Effect of sodium consumption on systolic blood pressure	Systolic blood pressure change	Meta-analysis/meta-regression of 103 trials	Only trials with duration > 7 days were analysed.	Mozaffarian D, Fahimi S, Singh GM, *et al.* Global sodium consumption and death from cardiovascular causes. New England Journal of Medicine 2014;371(7):624–34.
Setting reference level of sodium consumption	Ideal sodium consumption below which no risk was considered	Evidence from ecologic studies randomised trials and meta-analyses of prospective cohort studies	Intake levels associated with the lowest risk ranged from 614 to 2391 mg/day. In large, well-controlled, randomised feeding trials, the lowest tested intake for which blood pressure	Mozaffarian D, Fahimi S, Singh GM, *et al.* Global sodium consumption and death from cardiovascular causes. New England Journal of Medicine 2014;371(7):624–34.

			reductions were clearly documented was 1500 mg/day.	
Relative risk for systolic blood pressure	CHD and stroke (ICD10: I20–I25 and I60–I69)	Pooled analysis of two individual-level meta-analysis	Stratified by age and sex. Adjusted for regression dilution and total blood cholesterol and, where available, lipid fractions (HDL and non-HDL cholesterol), diabetes, weight, alcohol consumption, and smoking at baseline.	Micha R, Peñalvo JL, Cudhea F, Imamura F, Rehm CD, Mozaffarian D. Association between dietary factors and mortality from heart disease, stroke, and type 2 diabetes in the United States. JAMA 2017;317(9):912–24.
	Any other mortality (excluding CHD and stroke)	Individual-level meta-analysis of 48 prospective cohort studies	Adjusted for age, sex, race or ethnicity, deprivation, smoking, diabetes, inactivity, alcohol, obesity	Stringhini S, Carmeli C, Jokela M, *et al*. Socioeconomic status and the 25 × 25 risk factors as determinants of premature mortality: a multicohort study and meta-analysis of 1·7 million men and women. The Lancet 2017;389(10075):1229–37
Setting reference level	Ideal systolic blood pressure	Evidence from randomised trials of	There may be health benefits by lowering systolic blood pressure	Singh GM, Danaei G, Farzadfar F, *et al*. The age-specific quantitative effects of metabolic

of systolic blood pressure[1]	below which no risk was considered	antihypertensive drugs and the Intersalt study	down to 110mmHg	risk factors on cardiovascular diseases and diabetes: a pooled analysis. PLOS ONE 2013;8(7):e65174.|
Disease costs	Hospitalisation costs for CHD and stroke	Based on the Medical Expenditure Panel Survey (MEPS)	Stratified by age and sex	Ministry of Health, National Hospital Information System (SIH/SUS – *Sistema de Informações Hospitalares*) - Underlying cause of hospitalization based on the ICD-10 codes. 2018
	Informal care costs for CHD and stroke		Costs were extrapolated for Brazilian settings	Leal J, Luengo-Fernández R, Gray A, Petersen S, Rayner M. Economic burden of cardiovascular diseases in the enlarged European Union. Eur Heart J 2006;27(13):1610–9.

Table S2 Key modelling assumptions and limitations.

Population module
We assumed no migration after the age of 30
We assumed POF and PNS to be representative of the Brazilian population
Disease module
We assumed a log-linear exposure-response relationship for SBP with a 5-year mean lag time
We only modelled the first-ever event of CHD and stroke because we focus on primary prevention
For CHD and stroke initial incidence rates (year 2018), we used modelled estimates derived from mortality and PNS 2013 prevalence data
We assumed the non-attributable to SBP incidence rate trends for CHD and stroke to be 50% of the forecasted mortality rates trends
We assumed that the risk ratios of SBP on CHD and stroke incidence and mortality are equal and SBP is not modifying CHD and stroke survival
We assumed that changes in sodium consumption have an immediate effect on SBP and changes in SBP have a median 5-year time lag to impact the risk of CVD
Policy module
We assumed that the recently observed trends in sodium consumption, SBP, and disease-specific mortality would continue in the future (baseline scenario)
We assumed that the Brazilian population diet has and will have similar food composition since 2017-18
We assumed a full compliance to all policies by food industries in all regulatory policy scenarios
We assumed that changes in the sodium content of foods would be immediate after policy implementation
We assumed that individuals would not change sodium consumption behaviour because of sodium reduction in the foods.

Table S3. Priority food categories in the Brazilian sodium target agreements, the equivalent food categories in the POF surveys and their mean sodium content at the baseline in 2017 (mg/100g).

Food description sodium target agreements	POF Food category	Baseline
Instant pasta (noodles)	Massas instantâneas	1598.6
Industrialized sliced bread	Pão de forma	365.0
Buns	N/A	374.4
Cakes without filling	Bolo sem recheio	241.1
Cakes with filling	N/A	185.8
Creamy cake mixes[a]	Mistura para bolo	229.6
Aerated cake mixes[a]	Mistura para bolo	291.6
Potato chips	Batata frita, batata palha	475.4
Extruded corn snacks	Snacks	827.4
Filled cookies	Biscoito recheado	235.5
Salted crackers	Biscoito salgado	590.9
Sweet biscuits	Biscoitos doces	293.9
Mayonnaise	Maionese	852.7
Dairy/cheese spread	Requeijão	434.5
Margarines	Margarina	544.3
Mozzarella cheese	Queijo muçarela	517.2
Rice condiments	Demais temperos	31,260.0
Bouillon cubes or powders[b]	Caldo cubo, pó	952.1
Paste condiments	Tempero em pasta	31,845.7
Breakfast cereals	Cereais matinais	359.2
Soups	Sopa	295.1
Breaded meat	Empanados	588.7
Hotdog	Salsicha	1,082.9
Bologne	Mortadela	1,435.5

Sausage	Linguiça cozida	1,210.8
Hamburger	Hambúrguer	630.2
Fresh sausage	Linguiça frescal	1,001.2
Ham	Presuntaria	1,186.6
Sausage (stored at room temperature)	Linguiça cozida	1,323.7
French bread	Pão francês	289.0

[a] = as consumed

[b]= prepared according to label instructions

Table S4. Lowest sodium content in food categories in 2019 (mg/100g).

Food category	Brazil	PAHO	UK	FDA/USA	Argentina	South Africa
Buns	388	400	450		501	380
Sliced bread	450	400	450	410	530	380
Instant pasta	1920	1333	350	1810		800
Cakes without fillings	332	205	280	270		
Cakes with fillings	242	205	280	270		
Cake roll	204	205	280	270		
Aerated cake mix	398	205	280	720		
Creamy cake mix	395	205	280	720		
Corn snacks	852	530	800	900		
Potato chips	586	530	580	480		550
Mayonnaise	1051	670	500	610		
Cookies	359	265	380	330	512	
Crackers	699	700	700	760	941	
Filled cookies	265		380		429	
Breakfast cereals	418	500	400	560		400
French bread			360/450			
Margarine	715		550	690		450
Liquid/gel bouillons	865					
Powder/cube bouillons	1025	18000	380	390	430	13000
Rice seasoning	32076	9100				
Paste seasonings	33134					
Other seasonings	21775	21775				
Mozzarella cheese	512		615	750		
Cheese spread	541	500	720	410		
Frescal sausage	970		550	680		600

Resfriated sausage	1210	690	550	950	1196	
Room temperature sausage	1500	1350				
Hamburger	740			550	850	
Hamburger	1160	690	650	1120	1196	850
Soups	314	306	250	1810	352	3500
Instant soups	330	306	250	1810	352	
Breaded meat	650	470		670	736	
Hotdog sausage	1120	690	750	1000	1196	
Resfriated mortadella	1180	690	650	1000		
Room temperature mortadella	1350	1350				
Cured processed meat						850
Uncured processed meat						650
Savoury snacks (non-salt flavour)						700
Savoury snacks (salt flavour)			1000			550
Bacon			1150	1780		
Canned sausages			700	690		
Cheddar hard cheeses			800	625		
Fresh chesse			270	380		
Cottage cheese			210	380		
Manteiga com sal			670	620		
Pizza			500	490		
Sandwich high salt fillings			600	560		
Sandwich without high salt fillings			350	490		
Ketchup			380	1050		
Light mayonnaise			680			
Salad dressing			600	920		
Pasta sauces			370			
Canned tuna			360	390		

Other canned fish			600	390		
Canned vegetables			50	320		
Canned peas			180			
Meat free products			500			
Dried beverages (chocolate, capuccino)			60	320		
Olives				780		
Tomato based sauces				570		
Creamy sauces				430		

Table S5. Estimated sodium intake by age and sex groups and by dietary sodium sources considering food reformulation caused by the Brazilian voluntary target scenarios (2017).

	Added salt				Other sodium sources			
	Mean	SE	CI – 95%		Mean	SE	CI – 95%	
Men								
30-34y	6,312.18	183.22	5,953.07	6,671.30	6,062.69	175.98	5,717.77	6,407.61
35-39y	6,450.00	197.95	6,062.02	6,837.98	6,200.51	190.29	5,827.53	6,573.48
40-44y	5,966.45	166.95	5,639.21	6,293.69	5,716.96	159.97	5,403.40	6,030.51
45-49y	6,257.26	265.73	5,736.43	6,778.09	6,007.77	255.13	5,507.70	6,507.83
50-54y	5,575.10	192.52	5,197.75	5,952.45	5,325.60	183.91	4,965.14	5,686.07
55-59y	5,814.69	239.13	5,346.00	6,283.39	5,565.20	228.87	5,116.61	6,013.79
60-64y	5,738.10	271.43	5,206.09	6,270.11	5,488.60	259.63	4,979.72	5,997.48
65-69y	6,104.86	251.83	5,583.82	6,625.89	5,855.36	241.54	5,355.62	6,355.10
Women								
30-34y	5,344.74	146.89	5,056.84	5,632.64	5,095.24	140.03	4,820.78	5,369.71
35-39y	5,324.91	139.50	5,051.48	5,598.34	5,075.42	132.97	4,814.80	5,336.04
40-44y	5,139.15	132.50	4,879.45	5,398.85	4,889.66	126.06	4,642.57	5,136.74
45-49y	5,175.08	134.35	4,911.74	5,438.42	4,925.59	127.88	4,674.95	5,176.23
50-54y	5,309.76	195.51	4,926.56	5,692.96	5,060.27	186.32	4,695.07	5,425.46
55-59y	5,080.89	156.52	4,774.10	5,387.68	4,831.40	148.84	4,539.67	5,123.12
60-64y	4,878.31	168.20	4,548.63	5,207.99	4,628.82	159.60	4,315.99	4,941.64
65-69y	5,403.88	187.61	5,036.15	5,771.60	5,154.39	178.95	4,803.64	5,505.13

Table S6. Estimated sodium intake by age and sex groups and by dietary sodium sources considering the mandatory implementation of the lowest global sodium targets in Brazil.

	Added salt				Other sodium sources			
	Mean	SE	CI – 95%		Mean	SE	CI – 95%	
Men								
30-34y	5,756.18	167.08	5,397.07	6,115.30	5,506.69	159.84	5,161.77	5,851.61
35-39y	5,894.00	180.89	5,506.02	6,281.98	5,644.51	173.23	5,271.53	6,017.48
40-44y	5,410.45	151.39	5,083.21	5,737.69	5,160.96	144.41	4,847.40	5,474.51
45-49y	5,701.26	242.12	5,180.43	6,222.09	5,451.77	231.52	4,951.70	5,951.83
50-54y	5,019.10	173.32	4,641.75	5,396.45	4,769.60	164.71	4,409.14	5,130.07
55-59y	5,258.69	216.26	4,790.00	5,727.39	5,009.20	206.00	4,560.61	5,457.79
60-64y	5,182.10	245.13	4,650.09	5,714.11	4,932.60	233.33	4,423.72	5,441.48
65-69y	5,548.86	228.89	5,027.82	6,069.89	5,299.36	218.60	4,799.62	5,799.10
Women								
30-34y	4,788.74	131.61	4,500.84	5,076.64	4,539.24	124.75	4,264.78	4,813.71
35-39y	4,768.91	124.93	4,495.48	5,042.34	4,519.42	118.40	4,258.80	4,780.04
40-44y	4,583.15	118.16	4,323.45	4,842.85	4,333.66	111.73	4,086.57	4,580.74
45-49y	4,619.08	119.92	4,355.74	4,882.42	4,369.59	113.44	4,118.95	4,620.23
50-54y	4,753.76	175.04	4,370.56	5,136.96	4,504.27	165.85	4,139.07	4,869.46
55-59y	4,524.89	139.39	4,218.10	4,831.68	4,275.40	131.71	3,983.67	4,567.12
60-64y	4,322.31	149.03	3,992.63	4,651.99	4,072.82	140.43	3,759.99	4,385.64
65-69y	4,847.88	168.31	4,480.15	5,215.60	4,598.39	159.65	4,247.64	4,949.13

Table S7. Estimated sodium intake by age and sex groups and by dietary sodium sources considering the consumption of 10% potassium salt in Brazil.

	Added salt				Other sodium sources			
	Mean	SE	CI – 95%		Mean	SE	CI – 95%	
Men								
30-34y	7,674.42	168.31	7,344.52	8,004.33	6,312.18	183.22	5,953.07	6,671.30
35-39y	7,727.56	192.82	7,349.62	8,105.49	6,450.00	197.95	6,062.02	6,837.98
40-44y	7,712.25	175.23	7,368.80	8,055.70	5,966.45	166.95	5,639.21	6,293.69
45-49y	7,312.35	163.53	6,991.83	7,632.87	6,257.26	265.73	5,736.43	6,778.09
50-54y	7,671.73	199.76	7,280.19	8,063.27	5,575.10	192.52	5,197.75	5,952.45
55-59y	7,095.57	191.30	6,720.62	7,470.51	5,814.69	239.13	5,346.00	6,283.39
60-64y	6,965.55	203.01	6,567.64	7,363.47	5,738.10	271.43	5,206.09	6,270.11
65-69y	6,586.52	271.70	6,024.38	7,148.66	6,104.86	251.83	5,583.82	6,625.89
Women								
30-34y	5,378.36	137.51	5,108.84	5,647.88	5,344.74	146.89	5,056.84	5,632.64
35-39y	5,402.00	109.97	5,186.45	5,617.54	5,324.91	139.50	5,051.48	5,598.34
40-44y	5,401.88	103.12	5,199.76	5,604.00	5,139.15	132.50	4,879.45	5,398.85
45-49y	5,141.73	113.49	4,919.28	5,364.18	5,175.08	134.35	4,911.74	5,438.42
50-54y	5,089.95	143.58	4,808.53	5,371.37	5,309.76	195.51	4,926.56	5,692.96
55-59y	5,213.10	134.97	4,948.56	5,477.64	5,080.89	156.52	4,774.10	5,387.68
60-64y	5,136.61	150.62	4,841.38	5,431.83	4,878.31	168.20	4,548.63	5,207.99
65-69y	4,780.91	165.98	4,455.57	5,106.24	5,403.88	187.61	5,036.15	5,771.60

Table S8. Estimated sodium intake by age and sex groups and by dietary sodium sources considering the implementation of mandatory front-of-package nutritional labelling in Brazil.

	Added salt				Other sodium sources			
	Mean	SE	CI – 95%		Mean	SE	CI – 95%	
Men								
30-34y	6119.18	177.62	5760.07	6478.30	5869.69	170.38	5524.77	6214.61
35-39y	6257.00	192.03	5869.02	6644.98	6007.51	184.37	5634.53	6380.48
40-44y	5773.45	161.55	5446.21	6100.69	5523.96	154.57	5210.40	5837.51
45-49y	6064.26	257.53	5543.43	6585.09	5814.77	246.93	5314.70	6314.83
50-54y	5382.10	185.86	5004.75	5759.45	5132.60	177.25	4772.14	5493.07
55-59y	5621.69	231.19	5153.00	6090.39	5372.20	220.93	4923.61	5820.79
60-64y	5545.10	262.30	5013.09	6077.11	5295.60	250.50	4786.72	5804.48
65-69y	5911.86	243.87	5390.82	6432.89	5662.36	233.58	5162.62	6162.10
Women								
30-34y	5151.74	141.59	4863.84	5439.64	4902.24	134.73	4627.78	5176.71
35-39y	5131.91	134.44	4858.48	5405.34	4882.42	127.91	4621.80	5143.04
40-44y	4946.15	127.52	4686.45	5205.85	4696.66	121.08	4449.57	4943.74
45-49y	4982.08	129.34	4718.74	5245.42	4732.59	122.87	4481.95	4983.23
50-54y	5116.76	188.40	4733.56	5499.96	4867.27	179.21	4502.07	5232.46
55-59y	4887.89	150.57	4581.10	5194.68	4638.40	142.89	4346.67	4930.12
60-64y	4685.31	161.55	4355.63	5014.99	4435.82	152.95	4122.99	4748.64
65-69y	5210.88	180.91	4843.15	5578.60	4961.39	172.25	4610.64	5312.13

Additional results from the main analysis

Table S9 Health-related model estimates for scenario 1 (voluntary salt limits) over the 20-year simulation period from 2019 to 2038 for Brazilian adults aged 30 to 79 years by sex. Values are the median estimate (95% UI).

**Outcome**	**Men**			**Women**			**Persons**		
**Cases prevented or postponed (CPP)**									
CHD	*Median*	*(95%UI)*		*Median*	*(95%UI)*		*Median*	*(95%UI)*	
30-49y	15,000	2,800	50,000	3,000	0	15,000	20,000	3,100	62,000
50-69y	62,000	18,000	130,000	22,000	5,500	52,000	83,000	27,000	180,000
≥70y	22,000	3,900	46,000	19,000	6,000	41,000	40,000	14,000	80,000
All ages	100,000	29,000	220,000	46,000	14,000	96,000	150,000	46,000	310,000
Stroke	*Median*	*(95%UI)*		*Median*	*(95%UI)*		*Median*	*(95%UI)*	
30-49y	7,500	740	22,000	4,900	0	20,000	13,000	2,800	41,000
50-69y	41,000	14,000	92,000	32,000	12,000	86,000	70,000	29,000	160,000
≥70y	21,000	6,300	43,000	21,000	5,000	46,000	42,000	13,000	91,000
All ages	69,000	24,000	150,000	60,000	23,000	140,000	130,000	49,000	270,000
**Deaths prevented or postponed (DPP)**									
CHD	*Median*	*(95%UI)*		*Median*	*(95%UI)*		*Median*	*(95%UI)*	
30-49y	250	0	740	0	0	250	250	0	740
50-69y	740	0	2,000	250	0	1,200	1,200	0	2,800
≥70y	250	-250	980	490	-250	1,500	740	0	2,000
All ages	1,400	120	3,400	980	0	2,500	2,200	490	4,700
Stroke	*Median*	*(95%UI)*		*Median*	*(95%UI)*		*Median*	*(95%UI)*	
30-49y	0	0	740	0	0	740	250	0	980
50-69y	980	120	2,100	490	0	1,700	1,500	360	3,600
≥70y	740	0	2,200	490	-250	1,600	1,200	120	3,300
All ages	1,700	360	3,700	1,200	250	3,000	3,100	1,100	5,900
Non-CVD	*Median*	*(95%UI)*		*Median*	*(95%UI)*		*Median*	*(95%UI)*	
30-49y	740	0	2,300	250	0	740	1,200	250	2,700
50-69y	4,200	1,300	6,800	2,700	980	5,000	6,900	2,700	11,000
≥70y	3,700	1,100	6,500	3,400	1,500	7,100	7,100	3,400	13,000
All ages	8,600	4,700	15,000	6,400	3,300	12,000	15,000	8,700	26,000
Total	12,000	7,500	18,000	8,600	5,200	15,000	21,000	14,000	33,000

Table S10 Health-related model estimates over the 20-year simulation period, from 2019 to 2038, of the mandatory implementation of the lowest global sodium targets (scenario 2) for Brazilian adults aged 30 to 79 years by sex. Values are the median estimate (95% UI).

**Outcome**	**Men**			**Women**			**Persons**		
**Cases prevented or postponed (CPP)**									
CHD	*Median*	*(95%UI)*		*Median*	*(95%UI)*		*Median*	*(95%UI)*	
30-49y	37,000	8,300	95,000	8,000	120	26,000	46,000	9,000	120,000
50-69y	130,000	33,000	240,000	48,000	12,000	130,000	180,000	47,000	370,000
≥70y	45,000	12,000	86,000	41,000	14,000	92,000	86,000	31,000	170,000
All ages	210,000	54,000	420,000	93,000	30,000	230,000	310,000	86,000	640,000
Stroke	*Median*	*(95%UI)*		*Median*	*(95%UI)*		*Median*	*(95%UI)*	
30-49y	15,000	3,700	41,000	10,000	1,400	35,000	26,000	6,500	69,000
50-69y	83,000	29,000	190,000	74,000	25,000	160,000	150,000	56,000	330,000
≥70y	43,000	16,000	99,000	42,000	13,000	91,000	87,000	33,000	190,000
All ages	140,000	49,000	320,000	130,000	42,000	270,000	260,000	94,000	580,000
**Deaths prevented or postponed (DPP)**									
CHD	*Median*	*(95%UI)*		*Median*	*(95%UI)*		*Median*	*(95%UI)*	
30-49y	490	0	1,200	0	0	490	490	0	1,600
50-69y	1,700	250	3,400	740	0	2,000	2,500	740	5,200
≥70y	980	-380	1,700	980	-130	2,300	1,700	250	3,700
All ages	3,000	850	6,000	1,700	120	3,700	4,700	1,600	9,000
Stroke	*Median*	*(95%UI)*		*Median*	*(95%UI)*		*Median*	*(95%UI)*	
30-49y	250	0	980	250	0	980	490	0	1,500
50-69y	2,000	610	3,800	1,200	360	2,700	3,200	1,500	5,800
≥70y	1,500	250	3,400	1,200	250	3,300	2,700	740	5,500
All ages	3,400	1,600	7,000	2,700	970	5,800	6,500	3,100	12,000
Non-CVD	*Median*	*(95%UI)*		*Median*	*(95%UI)*		*Median*	*(95%UI)*	
30-49y	2,000	740	4,900	490	0	1,500	3,000	980	5,700
50-69y	11,000	5,200	19,000	6,600	2,700	12,000	18,000	9,000	31,000
≥70y	10,000	5,300	17,000	10,000	5,200	16,000	20,000	11,000	33,000
All ages	23,000	13,000	39,000	17,000	9,300	29,000	40,000	22,000	66,000
Total	30,000	19,000	47,000	22,000	13,000	34,000	52,000	32,000	80,000

Table S11 Health-related model estimates over the 20-year simulation period, from 2019 to 2038, of mandatory front-of-pack nutritional warnings in addition to voluntary limits (scenario 3) for Brazilian adults aged 30 to 79 years by sex. Values are the median estimate (95% UI).

Outcome	Men			Women			Persons		
**Cases prevented or postponed (CPP)**									
CHD	*Median*	*(95%UI)*		*Median*	*(95%UI)*		*Median*	*(95%UI)*	
30-49y	21,000	4,000	74,000	4,400	0	20,000	29,000	3,900	90,000
50-69y	87,000	24,000	190,000	34,000	6,300	78,000	120,000	34,000	260,000
≥70y	31,000	8,200	64,000	27,000	10,000	63,000	59,000	20,000	120,000
All ages	140,000	41,000	320,000	66,000	23,000	150,000	210,000	69,000	460,000
Stroke	*Median*	*(95%UI)*		*Median*	*(95%UI)*		*Median*	*(95%UI)*	
30-49y	10,000	1,200	33,000	7,400	120	30,000	19,000	4,100	59,000
50-69y	59,000	21,000	130,000	47,000	19,000	130,000	100,000	44,000	250,000
≥70y	29,000	9,400	64,000	30,000	8,400	72,000	64,000	19,000	130,000
All ages	99,000	36,000	210,000	84,000	33,000	220,000	190,000	68,000	410,000
**Deaths prevented or postponed (DPP)**									
CHD	*Median*	*(95%UI)*		*Median*	*(95%UI)*		*Median*	*(95%UI)*	
30-49y	250	0	1,100	0	0	490	250	0	1,200
50-69y	1,200	0	3,600	490	-130	1,500	2,000	0	4,400
≥70y	490	-490	1,600	740	-250	2,500	980	-130	3,300
All ages	2,000	-130	5,300	1,200	-130	3,900	3,200	480	7,300
Stroke	*Median*	*(95%UI)*		*Median*	*(95%UI)*		*Median*	*(95%UI)*	
30-49y	0	0	1,200	0	0	980	250	0	1,700
50-69y	1,200	250	3,600	740	0	2,600	2,200	610	5,300
≥70y	980	0	3,500	860	-250	2,700	2,000	0	5,200
All ages	2,700	490	5,300	2,000	360	4,900	4,700	1,600	8,900
Non-CVD	*Median*	*(95%UI)*		*Median*	*(95%UI)*		*Median*	*(95%UI)*	
30-49y	1,200	0	3,400	250	0	1,400	2,000	490	4,200
50-69y	6,900	2,600	11,000	4,300	1,600	8,600	11,000	4,500	18,000
≥70y	6,100	2,300	10,000	5,400	2,800	12,000	12,000	6,000	21,000
All ages	14,000	8,000	25,000	10,000	5,300	20,000	25,000	14,000	41,000
Total	19,000	12,000	30,000	14,000	8,000	24,000	33,000	20,000	54,000

Table S12. Health-related model estimates over the 20-year simulation period (2019 to 2038) for the universal use of 10% potassium salt, in addition to voluntary limits (scenario 4), among Brazilian adults aged 30 to 79 years, by sex. Values are the median estimate (95% UI).

**Outcome**	**Men**			**Women**			**Persons**		
**Cases prevented or postponed (CPP)**									
CHD	*Median*	*(95%UI)*		*Median*	*(95%UI)*		*Median*	*(95%UI)*	
30-49y	54,000	15,000	140,000	8,900	0	30,000	65,000	18,000	160,000
50-69y	170,000	59,000	350,000	56,000	15,000	120,000	230,000	71,000	470,000
≥70y	64,000	17,000	110,000	45,000	19,000	96,000	110,000	36,000	200,000
All ages	300,000	110,000	580,000	110,000	41,000	230,000	400,000	140,000	820,000
Stroke	*Median*	*(95%UI)*		*Median*	*(95%UI)*		*Median*	*(95%UI)*	
30-49y	27,000	8,300	63,000	19,000	3,800	48,000	48,000	14,000	100,000
50-69y	140,000	55,000	270,000	95,000	35,000	190,000	240,000	97,000	440,000
≥70y	80,000	30,000	140,000	63,000	22,000	130,000	150,000	52,000	260,000
All ages	260,000	96,000	460,000	180,000	63,000	360,000	440,000	170,000	790,000
**Deaths prevented or postponed (DPP)**									
CHD	*Median*	*(95%UI)*		*Median*	*(95%UI)*		*Median*	*(95%UI)*	
30-49y	740	0	2,700	0	0	740	740	0	2,800
50-69y	7,400	2,800	12,000	3,000	250	4,700	11,000	3,400	17,000
≥70y	4,900	1,500	9,700	3,400	250	6,100	8,400	2,100	16,000
All ages	14,000	5,200	24,000	6,600	970	11,000	20,000	6,700	33,000
Stroke	*Median*	*(95%UI)*		*Median*	*(95%UI)*		*Median*	*(95%UI)*	
30-49y	1,700	0	4,100	490	0	2,800	2,200	0	6,500
50-69y	12,000	5,000	19,000	6,900	2,600	11,000	19,000	8,100	29,000
≥70y	12,000	4,800	21,000	8,400	3,100	14,000	21,000	7,700	35,000
All ages	27,000	10,000	43,000	15,000	6,300	27,000	43,000	16,000	70,000
Non-CVD	*Median*	*(95%UI)*		*Median*	*(95%UI)*		*Median*	*(95%UI)*	
30-49y	2,600	610	5,900	490	0	1,600	3,200	980	6,800
50-69y	12,000	5,700	20,000	6,100	2,300	12,000	18,000	9,100	32,000
≥70y	11,000	5,800	19,000	8,200	4,100	16,000	20,000	11,000	33,000
All ages	26,000	14,000	42,000	15,000	7,500	29,000	41,000	23,000	69,000
Total	67,000	43,000	91,000	38,000	23,000	55,000	110,000	67,000	140,000

References

1. Pearson-Stuttard J, Kypridemos C, Collins B, Mozaffarian D, Huang Y, Bandosz P, et al. Estimating the health and economic effects of the proposed US Food and Drug Administration voluntary sodium reformulation: Microsimulation cost-effectiveness analysis. PLoS Med [Internet]. 2018;15(4):e1002551. Available from: https://doi.org/10.1371/journal.pmed.1002551

2. Pearson-Stuttard J, Kypridemos C, Collins B, Mozaffarian D, Huang Y, Bandosz P, et al. Estimating the health and economic effects of the proposed US Food and Drug Administration voluntary sodium reformulation: Microsimulation cost-effectiveness analysis. PLOS Med. 2018 Apr;15(4):e1002551.

3. Laverty AA, Kypridemos C, Seferidi P, Vamos E, Pearson-Stuttard J, Collins B, et al. Quantifying the impact of the Public Health Responsibility Deal on salt intake, cardiovascular disease and gastric cancer burdens: interrupted time series and microsimulation study. J Epidemiol Community Health [Internet]. 2018;73(9):881–7. Available from: https://doi.org/10.1136/jech-2018-211749

4. Kypridemos C, Guzman-Castillo M, Hyseni L, Hickey GL, Bandosz P, Buchan I, et al. Estimated reductions in cardiovascular and gastric cancer disease burden through salt policies in England: An IMPACT NCD microsimulation study. BMJ Open [Internet]. 2017;7(1):e013791. Available from: https://doi.org/10.1136/bmjopen-2016-013791

5. IBGE. Brazilian Population Estimates [Internet]. 2017. Available from: https://www.ibge.gov.br/en/statistics/social/population/18448-population-estimates.html?=&t=o-que-e

6. IBGE. Aquisição Alimentar Domiciliar Per Capita - Pesquisa de Orçamentos Familiares - POF 2008-2009 [Internet]. IBGE - Instituto Brasileiro de Geografia e Estatistica; 2010. Available from: https://biblioteca.ibge.gov.br/visualizacao/livros/liv47307.pdf

7. IBGE. Pesquisa Nacional de Saúde - PNS 2013 [Internet]. Pesquisa Nacional de Saúde - PNS 2013. 2014. Available from: https://www.ibge.gov.br/estatisticas/sociais/saude/9160-pesquisa-nacional-de-saude.html?=&t=microdados

8. Stasinopoulos M, Rigby B, Akantziliotou C. Instructions on how to use the gamlss package in R [Internet]. 2nd ed. 2008. 206 p. Available from: http://www.gamlss.com/wp-

content/uploads/2013/01/gamlss-manual.pdf

9. Micha R, Peñalvo JL, Cudhea F, Imamura F, Rehm CD, Mozaffarian D. Association between dietary factors and mortality from heart disease, stroke, and type 2 diabetes in the United States. JAMA - J Am Med Assoc [Internet]. 2017;317(9):912–24. Available from: https://doi.org/10.1001/jama.2017.0947

10. Mozaffarian D, Clarke R. Quantitative effects on cardiovascular risk factors and coronary heart disease risk of replacing partially hydrogenated vegetable oils with other fats and oils. Eur J Clin Nutr [Internet]. 2009 [cited 2022 Jan 28];63 Suppl 2:S22–33. Available from: https://pubmed.ncbi.nlm.nih.gov/19424216/

11. Barendregt JJ, van Oortmarssen GJ, Vos T, Murray CJL. A generic model for the assessment of disease epidemiology: The computational basis of DisMod II. Popul Health Metr [Internet]. 2003;1(1):4. Available from: https://doi.org/10.1186/1478-7954-1-4

12. Dong C, Bu X, Liu J, Wei L, Ma A, Wang T. Cardiovascular disease burden attributable to dietary risk factors from 1990 to 2019: a systematic analysis of the Global Burden of Disease Study. Nutr Metab Cardiovasc Dis. 2021 Nov 29;

13. Boshuizen HC, Lhachimi SK, van Baal PHM, Hoogenveen RT, Smit HA, Mackenbach JP, et al. The DYNAMO-HIA Model: An Efficient Implementation of a Risk Factor/Chronic Disease Markov Model for Use in Health Impact Assessment (HIA). Demography [Internet]. 2012;49:1259–83. Available from: https://doi.org/10/f4jzrb

14. Ministério da Saúde. SIM - Mortality Information System [Internet]. 2017. Available from: http://tabnet.datasus.gov.br/cgi/deftohtm.exe?sim/cnv/obt10uf.def

15. Hyndman RJ. Forecasting mortality, fertility, migration and population data [Internet]. 2017. Available from: http://cran.r-project.org/package=demography

16. Hyndman RJ, Booth H, Yasmeen F. Coherent Mortality Forecasting: The Product-Ratio Method With Functional Time Series Models. Demography [Internet]. 2013;50(1):261–83. Available from: https://doi.org/10.1007/s13524-012-0145-5

17. Stringhini S, Carmeli C, Jokela M, Avendaño M, Muennig P, Guida F, et al. Socioeconomic status and the 25 × 25 risk factors as determinants of premature mortality: a multicohort study and meta-analysis of 1·7 million men and women. Lancet [Internet]. 2017;389(10075):1229–37. Available from: https://doi.org/10.1016/S0140-6736(16)32380-7

18. Ministério da Saúde. SIH-SUS - Hospital Information System [Internet]. 2023. Available from: http://tabnet.datasus.gov.br/cgi/deftohtm.exe?sih/cnv/niuf.def

19. Leal J, Luengo-Fernández R, Gray A, Petersen S, Rayner M. Economic burden of cardiovascular diseases in the enlarged European Union. Eur Heart J [Internet]. 2006;27(13):1610–9. Available from: https://doi.org/10.1093/eurheartj/ehi733

20. Mozaffarian D, Fahimi S, Singh GM, Micha R, Khatibzadeh S, Engell RE, et al. Global sodium consumption and death from cardiovascular causes. N Engl J Med [Internet]. 2014;371(7):624–34. Available from: https://doi.org/10.1056/NEJMoa1304127

21. Ministério da Saúde. Agenda de Reformulação da Redução de Sódio, Açúcar e Gordura Trans da Promoção da Saúde e da Alimentação Adequada e Saudável. Agenda de Reformulação. 2023.

22. Public Health England. Salt Reduction Targets for 2017 [Internet]. 2017. Available from: https://assets.publishing.service.gov.uk/government/uploads/system/uploads/attachment_data/file/604338/Salt_reduction_targets_for_2017.pdf

23. South Africa. Regulations relating to the reduction of sodium in certain foodstuffs and related matters [Internet]. 2013. p. 8. Available from: https://extranet.who.int/ncdccs/Data/ZAF_B23_R214 of 20 March 2013 Sodium Reduction Regulations.pdf

24. Argentina. Resolución Conjunta 1/2018 [Internet]. Resolución Conjunta 1/2018. 2018 [cited 2020 Jan 24]. Available from: https://www.argentina.gob.ar/normativa/nacional/resolución-1-2018-317245/texto

25. FDA. Draft Guidance for Industry: Target Mean and Upper Bound Concentrations for Sodium in Commercially Processed, Packaged, and Prepared Foods for Voluntary Sodium Reduction Goals [Internet]. 2016. Available from: https://www.fda.gov/regulatory-information/search-fda-guidance-documents/draft-guidance-industry-target-mean-and-upper-bound-concentrations-sodium-commercially-processed

26. Campbell N, Legowski B, Legetic B, Nilson E, L'Abbé M. Inaugural Maximum Values for Sodium in Processed Food Products in the Americas. J Clin Hypertens [Internet]. 2015;17(8):611–3. Available from: https://doi.org/10.1111/jch.12553

27. Pan American Health Organization (PAHO). Pan American Health Organization Nutrient Profile Model [Internet]. Washington, DC; 2016. 38 p. Available from:

https://iris.paho.org/handle/10665.2/18623

28. Khandpur N, Sato PDMLA, Martins APB, Spinillo, Carla Galvão Garcia MT, Rojas CFU, Jaime PC. Are Front-of-Package Warning Labels More Effective at Communicating Nutrition Information than Traffic-Light Labels? A Randomized Controlled Experiment in a Brazilian Sample. Nutrients [Internet]. 2018;10(6):688. Available from: https://doi.org/10.3390/nu10060688

29. D'Elia L, Barba G, Cappuccio FP, Strazzullo P. Potassium intake, stroke, and cardiovascular disease a meta-analysis of prospective studies. J Am Coll Cardiol [Internet]. 2011 Mar 8 [cited 2022 Jan 4];57(10):1210–9. Available from: https://pubmed.ncbi.nlm.nih.gov/21371638/

30. Steenland K, Armstrong B. An overview of methods for calculating the burden of disease due to specific risk factors. Epidemiology [Internet]. 2006;17(5):512–9. Available from: https://doi.org/10.1097/01.ede.0000229155.05644.43

31. He FJ, Li J, MacGregor GA. Effect of longer term modest salt reduction on blood pressure: Cochrane systematic review and meta-analysis of randomised trials. BMJ [Internet]. 2013;346:f1325. Available from: https://doi.org/10.1136/bmj.f1325
